# Supplementary material for: Randomised controlled trial of exercise to prevent shoulder problems in women undergoing breast cancer treatment: study protocol for the prevention of shoulder problems trial (UK PROSPER)
Source: BMJ Open. 2018 Mar 23;8(3):e019078. doi: 10.1136/bmjopen-2017-019078 (PMC5875650; doi:10.1136/bmjopen-2017-019078)
Supplement: Author's manuscript [file bmjopen-2017-019078.draft_revisions.pdf]

BMJ Open

BMJ Open is committed to open peer review. As part of this commitment we make the peer review history of every article we publish publicly available.

When an article is published we post the peer reviewers' comments and the authors' responses online. We also post the versions of the paper that were used during peer review. These are the versions that the peer review comments apply to.

The versions of the paper that follow are the versions that were submitted during the peer review process. They are not the versions of record or the final published versions. They should not be cited or distributed as the published version of this manuscript.

BMJ Open is an open access journal and the full, final, typeset and author-corrected version of record of the manuscript is available on our site with no access controls, subscription charges or pay-per-view fees (http://bmjopen.bmj.com).

If you have any questions on BMJ Open's open peer review process please email editorial.bmjopen@bmj.com

BMJ Open

A randomised controlled trial of exercise to prevent shoulder problems in women undergoing breast cancer treatment: study protocol for the Prevention of Shoulder Problems Trial (PROSPER)

Journal:	*BMJ Open*
Manuscript ID	bmjopen-2017-019078
Article Type:	Protocol
Date Submitted by the Author:	11-Aug-2017
Complete List of Authors:	Bruce, Julie; University of Warwick, Warwick Clinical Trials Unit<br>Williamson, Esther; Nuffield Department of Orthopaedics, Rheumatology and Musculoskeletal Sciences, University of Oxford, United Kingdom. , Centre for Rehabilitation Research in Oxford<br>Lait, Clare; Gloucestershire Care Services NHS Trust<br>Richmond, Helen ; University of Warwick, Warwick Clinical Trials Unit<br>Betteley, Lauren; University of Warwick Warwick Medical School, Warwick Clinical Trials Unit<br>Lall, Ranjit; University of Warwick, Warwick Clinical Trials Unit<br>Petrou, Stavros; University of Warwick, Warwick Medical School<br>Rees, Sophie; University of Warwick, Warwick Clinical Trials Unit<br>Lamb, Sarah; University of Oxford, Nuffield Department of Orthopaedics, Rheumatology and Musculoskeletal Sciences<br>Thompson, Alastair; University of Texas M.D. Anderson Cancer Center, Department of Breast Surgical Oncology and Department of Translational Molecular Pathology
Keywords:	Breast surgery < SURGERY, Clinical trials < THERAPEUTICS, Rehabilitation medicine < INTERNAL MEDICINE

SCHOLARONE™
Manuscripts

For peer review only - http://bmjopen.bmj.com/site/about/guidelines.xhtml

A randomised controlled trial of exercise to prevent shoulder problems in women undergoing breast cancer treatment: study protocol for the Prevention of Shoulder Problems Trial (PROSPER)

Authors: Julie Bruce[1], Esther Williamson[2], Clare Lait[3], Helen Richmond[1], Lauren Betteley[1], Ranjit Lall[1], Stavros Petrou[1], Sophie Rees[1], Sarah E Lamb[1,2] and Alastair Thompson[4] on behalf of the PROSPER Study Group

Author affiliations

1. Warwick Clinical Trials Unit, Division of Health Sciences, University of Warwick, Coventry CV4 7AL, UK
2. Nuffield Department of Orthopaedics Rheumatology & Musculoskeletal Sciences, University of Oxford, Windmill Road, Oxford, OX3 7LD, UK
3. Gloucestershire Care Services NHS Trust, 1010 Gloucester Business Park, Pioneer Avenue, Brockworth, Gloucester, GL3 4AW, UK
4. Department of Breast Surgical Oncology & Department of Translational Molecular Pathology, University of Texas M.D. Anderson Cancer Center, 1400 Pressler Drive, Houston, Texas, US

Corresponding author: *Professor Julie Bruce; julie.bruce@warwick.ac.uk*

For peer review only

For peer review only - http://bmjopen.bmj.com/site/about/guidelines.xhtml

Abstract (300 words)

Introduction

Musculoskeletal shoulder problems are common after breast cancer treatment. Early postoperative exercises targeting the upper limb may improve shoulder function. This protocol describes a National Institute for Health Research funded randomised controlled trial to evaluate the clinical and cost-effectiveness of an early supervised exercise programme compared to usual care, for women at high risk of developing shoulder problems after breast cancer surgery.

Methods and Analysis

This pragmatic two-armed, multicentre RCT is underway within secondary care in the United Kingdom. PROSPER aims to recruit 350 women from approximately 15 UK centres with follow-up at 6 weeks, 6 and 12 months after randomisation. Recruitment processes and intervention development were optimised through qualitative research during a six-month internal pilot phase. Participants are randomised to the PROSPER intervention or best practice usual care only. The PROSPER intervention is delivered by physiotherapists and incorporates three main components: shoulder-specific exercises targeting range of movement and strength; general physical activity; and behavioural strategies to encourage adherence and support exercise behaviour. The primary outcome is upper arm function assessed using the Disabilities of the Arm Shoulder and Hand (DASH) questionnaire at 12 months post-randomisation. Secondary outcomes include DASH subscales, acute and chronic pain, complications, health related quality of life, and healthcare resource use. We will interview a subsample of participants (n=10 intervention; n=10 usual care) to explore their experiences of the trial interventions.

Ethics and Dissemination

Ethical approval was granted from the NHS National Research Ethics Service Committee West Midlands (15/WM/0224) on 20.07.2015. The PROSPER study is the first multicentre UK clinical trial to investigate the clinical and cost-effectiveness of supported exercise in the prevention of shoulder problems in high risk women undergoing breast cancer surgery. The

For peer review only - http://bmjopen.bmj.com/site/about/guidelines.xhtml

findings will inform future clinical practice and provide valuable insight into the role of physiotherapy-supported exercise in breast cancer rehabilitation.

Trial registration: ISRCTN35358984

Protocol version: Version 2.1; dated 11/01/2017

Funding: NIHR HTA (Project Number 13/84/10)

Strengths and limitations of this study

Strengths:

- A large pragmatic study delivering a complex intervention to prevent postoperative health problems in newly diagnosed cancer patients within secondary care;
- A strength of the evaluation is the mixed methods approach incorporating embedded qualitative research and economic analysis

Limitations:

- Recruited participants undergo multiple cancer treatments thus experience a complicated postoperative recovery pathway.

For peer review only

For peer review only - http://bmjopen.bmj.com/site/about/guidelines.xhtml

Background

Breast cancer is the most common cancer in women in the UK with a 30% increase in incidence since the early 1970s (1). Due to advances in screening and treatment, the survival rate has progressively risen and now two thirds of women will survive for 20 years beyond their breast cancer diagnosis (1). The mainstay treatment is surgery to the breast and axilla, supplemented with chemotherapy, radiotherapy and endocrine therapy, depending upon tumour stage and other clinical criteria. As a consequence of these treatments, upper limb problems such as decreased shoulder range of movement (ROM), impaired strength, chronic pain, sensory disturbances and lymphoedema are common adverse treatment effects (2, 3). Studies suggest that up to 67% of women have arm or shoulder symptoms up to 3 years after treatment (4). Persistent upper limb dysfunction and pain are debilitating, have a negative impact upon sleep, quality of life, physical functioning and emotional wellbeing. Given successes in increasing survival, it is timely to identify strategies to improve the health-related quality of life of women after breast cancer treatment.

Several risk factors for shoulder and upper body problems after breast cancer treatment have been identified, including treatment-related factors, such as type of axillary surgery and radiotherapy (RT), and patient factors such as age, body mass index (BMI), and pre-existing shoulder problems (4, 5). Women undergoing mastectomy compared to breast conserving surgery are at greater risk of postoperative shoulder restrictions (odds ratio (OR) 5.7, 95% confidence interval (CI) 1.03, 31.2) (4). Additionally, those undergoing axillary lymph node clearance (ANC) are at greater risk of postoperative arm complaints compared to those having sentinel lymph node biopsy (SLNB; OR 9.8; 95% CI 3.5, 27.5) (4-6). Radiotherapy (RT), particularly to the axilla or chest wall, increases the odds of shoulder restriction (pooled OR 1.7, 95% CI 1.0, 2.9) and lymphoedema (pooled OR 1.5, 95% CI 1.2, 1.8) compared to women treated without adjuvant RT (4). Women reporting problems in the upper body and shoulder region before surgery are also at increased risk of chronic postoperative pain (5, 7).

For peer review only - http://bmjopen.bmj.com/site/about/guidelines.xhtml

BMI at time of surgery has been shown to have an independent negative effect on shoulder external rotation up to seven years after breast cancer treatment, and increased BMI is a risk factor for chronic postoperative pain and arm lymphoedema (7, 8). It is important that the UK National Health Service (NHS) provides optimal care for these women at high risk of developing shoulder problems to ensure recovery and return to usual activities after cancer treatment.

A Cochrane review identified 24 studies (2132 participants) investigating exercise following breast cancer surgery (9). Six studies (n=354), conducted outside of the UK, found that structured postoperative exercise significantly improved shoulder ROM in the short and long term when compared to usual care (9). Ten studies (n=1304) have evaluated timing of exercise delivery; programmes initiated immediately postoperatively (1-3 days) versus delayed exercise suggest that early postoperative exercise does significantly improve long-term shoulder ROM. However, some studies reported an increased risk of wound-related complications with early exercise, such as seroma and surgical site infection (9). The largest UK trial to date (n=116 patients), published after the Cochrane review, found that participants were less likely to develop lymphoedema when exercises were limited to 90° of shoulder elevation during the first postoperative week compared to those performing unrestricted exercises (10). These previous trials investigating the efficacy of exercise following breast cancer surgery have been criticised for being of poor methodological quality and for omitting important patient-reported outcomes such as function and health-related quality of life (9). Furthermore, there is ongoing uncertainty around the optimal type, dose, and timing of exercise after breast cancer treatment. Moreover, none of the trials conducted to date have investigated the cost-effectiveness of structured exercise programmes after breast cancer treatment.

For peer review only

Rationale for a trial

To date, no large scale, high-quality, multicentre RCT investigating the clinical effectiveness of a structured physiotherapy intervention for women undergoing breast cancer surgery has been conducted. Given the lack of knowledge regarding the intensity and duration of exercise interventions after breast cancer surgery, this trial will provide evidence on whether a rigorously designed physiotherapy-led intervention, incorporating behaviour change theory, improves postoperative function and related outcomes, and whether this is cost-effective to deliver in the NHS setting.

Methods

Aim

The overall aim of PROSPER is to investigate the clinical and cost-effectiveness of an early supervised exercise programme compared to best practice usual care for women at high risk of shoulder problems after treatment for breast cancer, on outcomes of upper arm function, complications and quality of life. Specific trial objectives are:

1. To develop and refine a complex intervention of physiotherapy-led exercise for women at risk of developing musculoskeletal problems after breast cancer treatment;
2. To assess the acceptability of the exercise programme and outcome measures, optimise participant recruitment and refine trial processes during a six-month internal pilot phase at three clinical centres;
3. Use findings from the internal pilot phase to undertake a definitive RCT in approximately 15 UK NHS breast cancer centres.

This protocol follows guidance from the Standard Protocol Items: Recommendations for Interventional Trials (SPIRIT)(11). Core trial information is presented in Table 1 (WHO Trial Registration Data Set). Figure 1 details the schedule of enrolment, interventions and assessment.

For peer review only - http://bmjopen.bmj.com/site/about/guidelines.xhtml

Trial design and setting

A multicentre, pragmatic, parallel, two-arm RCT with an internal pilot study and embedded economic evaluation and qualitative studies. The trial framework is superiority rather than equivalence or exploratory. The trial is currently open and recruiting from 17 NHS tertiary breast cancer centres across England. Participants are randomised in a 1:1 ratio between intervention and control arms.

Patient and public involvement (PPI) in trial design

Four female PPI representatives, all of whom were treated for breast cancer, were consulted during the initial grant preparation, intervention development and trial set up. Our PPI representatives contributed to the design of the intervention and advised on recruitment-related issues; they provided valuable insight into the worries and concerns experienced during cancer treatment.

Eligibility Criteria

Women are eligible to participate in PROSPER if they are: diagnosed with histologically confirmed invasive or non-invasive primary breast cancer scheduled for surgical excision; aged 18 years or over; can comply with the protocol; willing to provide written informed consent; and considered as being at high risk of developing postoperative shoulder problems (Table 2). This is a pragmatic trial and it is important that inclusion criteria reflect contemporary clinical practice. Therefore, women are also eligible where a later decision is made for postoperative radiotherapy (RT) to the axilla and/or supraclavicular region, thus changing their risk status from low to high. 'Late entry' women are eligible for the trial if the decision for postoperative RT is made within six weeks of surgery. Women who have had previous breast surgery (such as excision of a benign tumour or breast cyst) and those women who have had previous contralateral (opposite side) mastectomy, are eligible for invitation providing they fulfil high risk criteria for shoulder problems. Women having

For peer review only - http://bmjopen.bmj.com/site/about/guidelines.xhtml

immediate reconstruction or bilateral breast surgery are ineligible as the usual NHS postoperative care pathway often includes routine postoperative physiotherapy. Exclusion criteria are presented in Table 2.

Participant screening, recruitment and consent

Participants are screened and identified from multi-disciplinary team (MDT) meetings and preoperative breast/oncology clinic lists in secondary care. The initial screening process is undertaken by a member of the clinical team, research nurse or trained designee. Potentially eligible patients are approached by clinical or research staff and are given a Patient Information Sheet (PIS) with further explanation of the trial. Figure 2 summarises participant flow.

Allocation sequence generation and randomisation

Randomisation is based upon a computer-generated algorithm held and controlled centrally by the Warwick Clinical Trials Unit (WCTU) programming team, independent from the PROSPER trial team. The WCTU telephone randomisation service is used whereby randomisation occurs after eligibility and informed consent has been obtained. Concealment of allocation is maintained. An automated confirmation email of intervention allocation is generated to the study team. Randomisation is stratified by the following variables: (i) first versus repeat surgery; (ii) centre; and (iii) whether informed of the need for radiotherapy within six weeks of surgery. The first variable adjusts for the requirement for any additional surgery which may change risk status from low to high (e.g. second procedure ANC or reexcision of surgical margins). The second stratification variable ensures balanced allocation across each recruitment site. The third variable accounts for late entry to the trial, thus relates to the timing of intervention delivery and whether participants are randomised preoperatively (up to the day of surgery) or within the first six weeks postoperatively. Due to the nature of the study intervention, it is not possible to blind participants or treating physiotherapists to treatment allocation. However, outcome data collection, data entry, data

For peer review only - http://bmjopen.bmj.com/site/about/guidelines.xhtml

cleaning and interim statistical analyses are conducted without knowledge of treatment allocation (blinded).

Interventions

Control Arm: Usual Care

Participants allocated to the usual care arm receive best practice usual care in the form of written leaflets containing information about exercises, recovery after surgery, and treatments for breast cancer. During the pilot phase, different exercise information leaflets were reviewed and considered; we also consulted best practice guidance for written patient information materials (12). The most commonly used information leaflets were 'Exercises after Breast Cancer Surgery (BCC6)' and 'Your Operation and Recovery (BCC151)' published by Breast Cancer Care (BCC) (13). The BCC leaflets were selected because of content, style and clarity of presentation of information. These leaflets are given to patients before surgery by breast care nurses, or other healthcare professionals, depending upon local practice.

Intervention Arm: PROSPER exercise programme

Participants randomised to the active intervention receive usual care leaflets in addition to the PROSPER intervention: a structured individualised exercise programme, comprising a minimum of three face-to-face and maximum of six sessions or contacts with a physiotherapist. A detailed description of intervention development has been submitted for publication. In brief, the PROSPER programme comprises specific exercises targeting shoulder range of motion and upper arm muscle strength, general physical activity, and behavioural adherence strategies. The intervention is predominantly delivered in physiotherapy outpatient departments.

The first physiotherapy session is arranged seven to ten days after surgery, for assessment of shoulder ROM, pain, function, patients' goals and assessment of confidence to carry out prescribed exercises. Participants are prescribed an individually tailored home exercise

For peer review only - http://bmjopen.bmj.com/site/about/guidelines.xhtml

programme and provided with guidance on rehabilitation, management of postoperative complications and returning to general physical activity and/or work. The second appointment is between four to six weeks postoperatively to review progress and prescribe shoulder strengthening exercises. The programme is progressed by increasing exercise repetitions, sets and resistance. The third appointment is recommended for between 12 to 16 weeks postoperatively, for further progression to facilitate return to work, sport and hobbies. For women with later entry on the basis of postoperative radiotherapy, these timings will be slightly delayed, but the exercise programme should commence at the earliest opportunity, thus within six weeks of surgery.

As per development work with patient representatives, and to reflect the pragmatic trial design, three additional physiotherapy consultations are available on request. The timing and delivery of additional appointments, either via telephone or face-to-face, are flexible to account for on-going treatment, physiotherapist judgement and patient preference. Ideally the intervention will be completed within the first six months following surgery, but women can contact their physiotherapist for up to 12 months after randomisation. Thus any late treatment-related shoulder problems will be dealt with by the trial physiotherapist. Number and method of physiotherapy contacts will be closely monitored during the trial.

Outcomes

Figure 1 presents the study outcome measures and standardised assessment scales by assessment time point. Questionnaires are completed at baseline on recruitment, then at 6 weeks, 6 and 12 months after randomisation by post. The primary outcome is upper limb function at 12 months measured using the Disabilities of Arm, Shoulder and Hand (DASH) questionnaire. The DASH is a 30-item patient-reported outcome measure designed to capture difficulty in performing various upper arm activities (14). A single DASH score is generated, although psychometric assessment using discriminant content validation analysis has shown that the scale can be used to produce three health outcome sub-scores for

For peer review only - http://bmjopen.bmj.com/site/about/guidelines.xhtml

impairment, activity limitation and participation restriction, as per the WHO International Classification of Functioning Disability and Health (ICF) taxonomy (15). Secondary outcomes include health-related quality of life, DASH sub-scores, and surgical adverse events including pain (acute, chronic, neuropathic pain) surgical site infection and lymphoedema. Data on exercise/mobility are collected to allow comparisons in activity. Healthcare resource use is recorded for economic analyses.

Internal pilot study

A six-month internal pilot phase was conducted at three breast cancer units (Coventry, Oxford and Wolverhampton) to evaluate processes for patient identification, eligibility and refinement of recruitment estimates. The intended sample size for the internal pilot study was 30 participants, approximately 10% of the full sample. Acceptability of the PROSPER intervention was explored through qualitative research involving audio-recorded individual interviews with seven participants. Data from the pilot phase helped to refine recruitment and trial processes. Patients recruited to the pilot phase continue with the follow-up schedule and will be retained in the full trial analysis.

Sample Size

The PROSPER trial aims to recruit 350 patients, allocated in a 1:1 ratio. The sample size calculation is based on a Dutch trial of thirty women with breast cancer, randomised to physiotherapy over a three month period, reporting a between group difference of 7 points on the DASH at 6 months (16). At 80% power and $p<0.05$, this yields a target of 242 participants in total. Accounting for therapist effects, an intracluster coefficient (ICC) of 0.01 (yielding a design effect of 1.05), gives a target of 256 participants. The ICC estimate is based on our previous experience of exercise interventions in a range of musculoskeletal trials. We anticipate loss to follow up of less than 10% based on our previous clinical trials however, have inflated this to 25% to cover the possibility that numbers lost to follow up are greater than anticipated e.g. due to ongoing cancer treatment.

For peer review only - http://bmjopen.bmj.com/site/about/guidelines.xhtml

The study is powered to detect a 7 point difference on the DASH. Studies of rheumatological and orthopaedic populations have suggested that the minimally clinically important difference (MCID) for the DASH is 10, and that the between group difference for trials should be set at 10 (17). However, this fails to account for many of the eventualities that occur in pragmatic trials, notably that there is not a ‘no treatment’ control arm, and therefore that some of the control group may be exposed by serendipity to an intervention of similar intensity, particularly in a high risk population.

Data analysis

Statistical analysis will be intention-to-treat and will comply with the CONSORT guidelines. The primary outcome data will be summarised using mean, standard deviation, median and range values. The clustering effect will be assessed prior to analysis of the data. In the presence of a clustering effect, the primary outcome will be analysed using multi-level linear regression models. If there is negligible clustering effect, it will be analysed using ordinary linear regression models. In each case, the mean change from baseline (to 6 and 12 months) will be summarised for each of the treatment arms and differences between the interventions using unadjusted and adjusted (for age, type of surgery and radiotherapy) estimates. These mean changes and their 95% confidence intervals will be plotted graphically so that change can be assessed over the course of the study. Continuous secondary outcomes will be assessed in a similar way to the primary outcome. Categorical data will be analysed using random effect/ordinary logistic models, depending on the presence of a clustering effect.

A DASH score cannot be computed if there are more than three missing items. As a sensitivity analysis, the impact of missing data will be assessed using multiple imputation. The impact of non-compliance with the intervention will be examined using the complier average causal effect (CACE) analysis (18, 19). We have reviewed definitions of compliance

For peer review only - http://bmjopen.bmj.com/site/about/guidelines.xhtml

for CACE analyses used in other therapy trials (20, 21). Complete compliance with the PROSPER intervention is defined as having three or more contacts with the PROSPER therapist; an additional analysis will be undertaken to explore partial compliance, defined as less than three sessions. Analyses and template tables will be reported in a detailed statistical analysis plan for review and approval by the DMC, prior to final statistical analysis of the data. Planned sensitivity analyses include: a) the impact of low/high recruitment centres on clustering effect; and b) assessment of differences between date of randomisation and date of surgery across groups, as surgical trials vary in relation to timing of follow-up.

Economic Evaluation

The primary economic evaluation will be conducted from the NHS and personal social services (PSS) perspective (22) using the intention-to-treat approach (23). Data will be collected on the health and social service resources used in the treatment of each trial participant from randomisation to 12 months post-randomisation. Primary research methods will be used to estimate the costs of delivering the physiotherapy-led exercise programme, including development and training of accredited providers, the cost of delivering the individual sessions and participant monitoring activities. Broader resource utilisation will be captured through three main sources: (i) clinical data extraction forms; (ii) patient postal questionnaires at 6 months and 12 months post-randomisation; and if feasible within the trial timeline, (iii) routine health data sources from NHS Digital. Current UK unit costs, will be applied to each resource item to estimate costs in each trial arm. Health-related quality of life will be measured at baseline and at 6 and 12 months post-randomisation using the generic EuroQol EQ-5D-5L and SF-12 measures; national tariff sets will be used to generate quality-adjusted life-years (QALYs) (24-28).

An incremental cost-effectiveness analysis, expressed in terms of incremental cost per QALY gained, will be performed. Detailed methods of analysis will be pre-specified within a

For peer review only - http://bmjopen.bmj.com/site/about/guidelines.xhtml

health economics analysis plan approved by the trial team prior to analysis to ensure appropriate methods are used. Results will be presented using incremental cost-effectiveness ratios (ICERs) and cost-effectiveness acceptability curves generated via the net-benefit framework. A series of sensitivity analyses will be undertaken to explore the implications of uncertainty on the ICERs and to consider the broader issue of the generalisability of the study results. Due to the known limitations of within-trial economic evaluations (29), a decision-analytical model may be constructed to examine the longer term costs and outcomes beyond the end of the trial. Costs and outcomes beyond the first year will be discounted to present values (22) and probabilistic sensitivity analyses will be undertaken to explore the impact of uncertainty on the ICERs.

Qualitative sub-study

An embedded qualitative study will be undertaken to gain insight into the experiences of women participating in trial interventions. We will explore the acceptability of the exercise programme and compare and contrast experiences with women allocated to the control intervention.

Design of sub-study

In-depth, semi-structured interviews will be conducted and audio-recorded. Interview topic guides will be used to ensure similar areas are covered in each interview. Participants consenting to the main trial are asked to indicate willingness to take part in a future interview to explore postoperative experiences. A total of twenty interviews are planned, with ten women from each intervention arm. Purposive sampling will be used, striving for a mix of geographical location, age, employment status, socio-economic background and ethnicity.

Analysis

Interviews will be recorded, transcribed and analysed using a Framework Approach. A thematic framework will be developed using pre-determined themes plus new themes raised

For peer review only - http://bmjopen.bmj.com/site/about/guidelines.xhtml

by participants. The framework will be applied to the interview text and coded data will be arranged on a chart according to each theme identified. Themes will be examined with a view to providing explanations of the participants' experiences and understandings.

Data security and management

Participant data is stored on a secure database in accordance with the Data Protection Act (1998). A unique trial identification number is used on all participant communication. Clinical and patient forms are being checked for completeness and congruity before data entry onto the PROSPER trial database. Data will undergo additional checks to ensure consistency between data submitted and original paper forms. Trial documentation and data will be archived for at least ten years after completion of the trial in accordance with WCTU standard operating procedures.

Trial monitoring

The Trial Management Group (TMG) will oversee all aspects of design, delivery, quality assurance and data analysis. A Trial Steering Committee (TSC), with independent Chairperson, will monitor the trial at least once per year. An independent DMC will review trial progress, recruitment, protocol compliance and interim analysis of outcomes, annually or more frequently as requested. Recruitment data from the internal pilot study were reviewed by independent committees and by the funder to approve the launch of the main trial.

Adverse event management

For peer review only

A safety reporting protocol has been developed for related and unexpected serious adverse events (SAEs) and directly attributable adverse events (AEs). An AE is defined as any untoward medical occurrence in a subject which does not necessarily have a causal relationship with the intervention. Any adverse event that occurs whilst undertaking PROSPER exercises, either during an appointment, or whilst exercising unsupervised at home, require reporting to the trial team. The trial Chief Investigator, with input from the WCTU Quality Assurance team, determine whether AEs require reporting to the trial sponsor, DMC and Ethics Committee, in accordance with the full safety reporting protocol.

Research ethics approval

Ethical approval was granted from the NHS National Research Ethics Service (NRES) Committee West Midlands (Solihull) (15/WM/0224) on 20th July 2015. Site-specific approvals have been obtained from NHS Research, Development and Innovation departments.

Dissemination policy

The study team are committed to full disclosure of the results of the trial. Findings will be reported in accordance with CONSORT guidelines (30) and we aim to publish in high impact journals. Our patient representatives will assist with dissemination of study results through INVOLVE, other cancer patient groups and organisations including www.independentcancerpatientsvoice.org.uk. The funder will take no role in the analysis or interpretation of trial results.

Discussion

The PROSPER trial will be the largest UK RCT examining the effectiveness of an early, supervised exercise and behavioural support intervention for women at risk of developing shoulder problems after breast cancer surgery. Previous trials in this field have been

For peer review only - http://bmjopen.bmj.com/site/about/guidelines.xhtml

criticised for being of poor methodological quality and lacking in important outcome measures, such as patient-reported shoulder function and health-related quality of life. PROSPER will provide empirical data on whether a physiotherapy-led exercise programme is effective for reducing shoulder disability when delivered in a pragmatic NHS clinical setting. The design and development of this complex intervention was underpinned by multiple stages of work, in line with MRC guidance on the development of complex interventions.

Acknowledgements

We extend very grateful thanks to all the trial participants. We are also grateful to all the physiotherapy staff, surgical oncology teams, breast cancer nurses and research departments collaborating on this study.

Collaborators

PROSPER Study Group: Chief Investigator: Professor Julie Bruce. Co-investigators (Grant holders): Professor Sarah E Lamb, Dr Esther Williamson, Dr Ranjit Lall, Professor Stavros Petrou, Mr Alastair M Thompson, Dr John Williams and Dr Catherine Harkin (deceased).

Trial Coordination/Administration: Mrs Emma J Withers, Mrs Lauren Betteley, Mr Craig Turner, Mrs Loraine Chowdhury.

Senior Project Managers: Mrs Susie Hennings, Mrs Helen Higgins.

Research Fellows/Associates: Dr Helen Richmond, Mrs Clare Lait (Physiotherapist), Dr Sophie Rees (Qualitative), Mr Pankaj Mistry (Medical Statistics), Dr Alastair Canaway (Health Economics).

Patient representatives: Dr Catherine Harkin (Deceased), Mrs Marie van Laar, Mrs Lyn Ankcorn.

Surgical Leads: Miss Abigail Tomlins, Miss Raghavan Vidya, Ms Pankaj G Roy, Miss Kat McEvoy.

For peer review only - http://bmjopen.bmj.com/site/about/guidelines.xhtml

Intervention development: Mrs Clare Lait, Dr Esther Williamson, Dr Cynthia Srikesavan, Mrs Jane Moser, Dr Meredith Newman, Dr Sophie Rees, Mrs Lauren Betteley, Dr Helen Richmond, Dr Beth Fordham, Professor Sarah E Lamb and Professor Julie Bruce.

Data Programming team: Mr Ade Willis, Mr Henry Adjei.

Quality Assurance: Ms Claire Daffern.

Contributors: JB obtained study funding with support from SEL, EW, RL, SP, AMT and JW. JB, SEL, EW, CL, RL, AMT, LB, SR and SP participated in the design of the study. EJW and LB coordinate study administration, acquisition of trial data and administrative support (CT/LC). PM will undertake statistical analysis, under direction of RL, senior trial statistician. AC is responsible for health economic analysis, supported by SP, senior health economist. JB and HR drafted the manuscript. All authors critically revised the manuscript for intellectual content and approved the final manuscript. This trial protocol is published on behalf of the PROSPER Study Group.

Funding

The PROSPER trial is funded by the National Institute of Health Research Technology Assessment Programme (NiHR HTA), project number 13/84/10. The views expressed in this publication are those of the authors and not necessarily those of the NIHR or Department of Health. This project benefited from facilities funded by Birmingham Science City Translational Medicine Clinical Research and Infrastructure Trials Platform, with support from Advantage West Midlands (AWM). The trial sponsor is the University of Warwick and University Hospitals Coventry and Warwickshire NHS Trust.

Trial Registration:

International Standard Randomised Controlled Trial Number: ISRCTN 35358984.

Competing interests

One author provides private physiotherapy to cancer patients (CL).

For peer review only - http://bmjopen.bmj.com/site/about/guidelines.xhtml

Data sharing statement

The trial statisticians and iDMC will have access to the dataset for the analysis of trial outcomes. The CI will have access to the data and take full responsibility for the analysis and publication of results. Once the main analyses have been undertaken, data will be available to other investigators subject to approval of data analysis plans by the steering committee and compliance with the University of Warwick Standard Operating Procedures on Data Management and Sharing. We will comply with Data Sharing Policies that may be instituted by the Funder (NIHR) during the lifetime of the project. The full PROSPER intervention manual and related materials will be available for wider access on completion of the main trial, according to funder and institutional repository requirements.

Data Monitoring Committee: Professor Malcolm Reed (Chair), Dr Rhian Gabe, Dr Matthew Maddocks.

Trial Steering Committee: Professor Steven Duffy (Chair), Dr Anna Kirby, Dr Karen Robb.

We dedicate this article to Professor Adele Frances (Deceased) who served on the PROSPER TSC from 2015-2016.

For peer review only

For peer review only - http://bmjopen.bmj.com/site/about/guidelines.xhtml

References

1. CRUK. *Cancer Research UK: Breast Cancer Survival Statistics* [cited 2014. Available from: http://www.cancerresearchuk.org/cancer-info/cancerstats/types/breast/survival/.

2. Mejdahl MK, Andersen KG, Gärtner R, Kroman N, Kehlet H. Persistent pain and sensory disturbances after treatment for breast cancer: six year nationwide follow-up study. BMJ. 2013;346:f1865.

3. McNeely ML, Binkley JM, Pusic AL, Campbell KL, Gabram S, Soballe PW. A prospective model of care for breast cancer rehabilitation: postoperative and postreconstructive issues. Cancer. 2012;118(8 Suppl):2226-36.

4. Lee TS, Kilbreath SL, Refshauge KM, Herbert RD, Beith JM. Prognosis of the upper limb following surgery and radiation for breast cancer. Breast Cancer Res Treat. 2008;110(1):19-37.

5. Bruce J, Thornton AJ, Powell R, Johnston M, Wells M, Heys SD, et al. Psychological, surgical, and sociodemographic predictors of pain outcomes after breast cancer surgery: a population-based cohort study. Pain. 2014;155(2):232-43.

6. Kootstra JJ, Dijkstra PU, Rietman H, de Vries J, Baas P, Geertzen JH, et al. A longitudinal study of shoulder and arm morbidity in breast cancer survivors 7 years after sentinel lymph node biopsy or axillary lymph node dissection. Breast Cancer Res Treat. 2013;139(1):125-34.

7. Meretoja TJ, Andersen KG, Bruce J, Haasio L, Sipila R, Scott NW, et al. Clinical Prediction Model and Tool for Assessing Risk of Persistent Pain After Breast Cancer Surgery. J Clin Oncol. 2017;35(15):1660-7.

8. DiSipio T, Rye S, Newman B, Hayes S. Incidence of unilateral arm lymphoedema after breast cancer: a systematic review and meta-analysis. The Lancet Oncology. 2013;14(6):500-15.

For peer review only

For peer review only - http://bmjopen.bmj.com/site/about/guidelines.xhtml

9. McNeely ML, Campbell K, Ospina M, Rowe BH, Dabbs K, Klassen TP, et al. Exercise interventions for upper-limb dysfunction due to breast cancer treatment. The Cochrane database of systematic reviews. 2010(6):CD005211.

10. Todd J, Scally A, Dodwell D, Horgan K, Topping A. A randomised controlled trial of 0two programmes of shoulder exercise following axillary node dissection for invasive breast cancer. Physiotherapy. 2008;94(4):265-73.

11. Chan A-W, Tetzlaff JM, Gøtzsche PC, Altman DG, Mann H, Berlin JA, et al. SPIRIT 2013 explanation and elaboration: guidance for protocols of clinical trials. Bmj. 2013;346:e7586.

12. Department of Health (DH). Toolkit for producing patient information. London, The Department of Health; 2003.

13. BCC. Breast Cancer Campaign [Available from: http://www.breastcancercare.org.uk/.

14. Harrington S, Michener LA, Kendig T, Miale S, George SZ. Patient-reported upper extremity outcome measures used in breast cancer survivors: a systematic review. Archives of physical medicine and rehabilitation. 2014;95(1):153-62.

15. Dixon D, Johnston M, McQueen M, Court-Brown C. The Disabilities of the Arm, Shoulder and Hand Questionnaire (DASH) can measure the impairment, activity limitations and participation restriction constructs from the International Classification of Functioning, Disability and Health (ICF). BMC musculoskeletal disorders. 2008;9:114.

16. Beurskens CH, van Uden CJ, Strobbe LJ, Oostendorp RA, Wobbes T. The efficacy of physiotherapy upon shoulder function following axillary dissection in breast cancer, a randomized controlled study. BMC cancer. 2007;7:166.

17. Sorensen AA, Howard D, Tan WH, Ketchersid J, Calfee RP. Minimal clinically important differences of 3 patient-rated outcomes instruments. The Journal of hand surgery. 2013;38(4):641-9.

For peer review only - http://bmjopen.bmj.com/site/about/guidelines.xhtml

18. Dunn G, Maracy M, Tomenson B. Estimating treatment effects from randomized clinical trials with noncompliance and loss to follow-up: the role of instrumental variable methods. Statistical methods in medical research. 2005;14(4):369-95.

19. Perneger TV, Burnand B. A simple imputation algorithm reduced missing data in SF-12 health surveys. Journal of clinical epidemiology. 2005;58(2):142-9.

20. Knox CR, Lall R, Hansen Z, Lamb SE. Treatment compliance and effectiveness of a cognitive behavioural intervention for low back pain: a complier average causal effect approach to the BeST data set. BMC musculoskeletal disorders. 2014;15:17.

21. Williams MA, Williamson EM, Heine PJ, Nichols V, Glover MJ, Dritsaki M, et al. Strengthening And stretching for Rheumatoid Arthritis of the Hand (SARAH). A randomised controlled trial and economic evaluation. Health Technol Assess. 2015;19(19):1-222.

22. NICE. *Guide to the Methods of Technology Appraisal* 2013 [Available from: http://publications.nice.org.uk/guide-to-the-methods-of-technology-appraisal-2013-pmg9.

23. Hollis S, Campbell F. What is meant by intention to treat analysis? Survey of published randomised controlled trials. Bmj. 1999;319(7211):670-4.

24. Dolan P. Modeling valuations for EuroQol health states. Medical care. 1997;35(11):1095-108.

25. van Hout B, Janssen MF, Feng YS, Kohlmann T, Busschbach J, Golicki D, et al. Interim scoring for the EQ-5D-5L: mapping the EQ-5D-5L to EQ-5D-3L value sets. Value in health : the journal of the International Society for Pharmacoeconomics and Outcomes Research. 2012;15(5):708-15.

26. Herdman M, Gudex C, Lloyd A, Janssen M, Kind P, Parkin D, et al. Development and preliminary testing of the new five-level version of EQ-5D (EQ-5D-5L). Quality of life research : an international journal of quality of life aspects of treatment, care and rehabilitation. 2011;20(10):1727-36.

27. Brazier J, Roberts J, Deverill M. The estimation of a preference-based measure of health from the SF-36. Journal of health economics. 2002;21(2):271-92.

For peer review only - http://bmjopen.bmj.com/site/about/guidelines.xhtml

28. Devlin N, Shah K, Feng Y, Mulhern B, van Hout B. Valuing health-related quality of life: an EQ-5D-5L value set for England. 2016.

29. Sculpher MJ, Claxton K, Drummond M, McCabe C. Whither trial-based economic evaluation for health care decision making? Health economics. 2006;15(7):677-87.

30. Moher D, Schulz KF, Altman DG. The CONSORT statement: revised recommendations for improving the quality of reports of parallel-group randomised trials. Lancet. 2001;357(9263):1191-4.

For peer review only

For peer review only - http://bmjopen.bmj.com/site/about/guidelines.xhtml

Table 1 World Health Organisation Trial Registration Data Set

Data category	Information
Primary registry and trial identifying number	ISRCTN35358984
Date of registration in primary registry	Project Number 13/84/10
Secondary identifying numbers	HTA
Source of monetary or material support	National Institute for Health Research (NIHR) Health Technology Assessment (HTA)
Joint sponsor	University of Warwick / University Hospitals Coventry & Warwickshire NHS Trust
Contact for public queries	prosper@warwick.ac.uk
Contact for scientific queries	Prof Julie Bruce, Warwick Clinical Trials Unit, University of Warwick
Public Title	Exercise to prevent shoulder problems in patients undergoing breast cancer treatment
Scientific Title	The PRevention Of Shoulder ProblEms Study (PROSPER): a randomised controlled clinical trial comparing physiotherapy-led exercise versus usual care in women at high risk of shoulder problems after breast cancer surgery
Countries of recruitment	UK
Health condition or problem studied	Breast cancer
Interventions	Advice only: Breast Cancer Care leaflets<br>Comparator: Physiotherapy-led structured exercise programme incorporating behavioural

For peer review only - http://bmjopen.bmj.com/site/about/guidelines.xhtml

	strategies
Key inclusion and exclusion criteria	Age: 18 years or over, no upper age restriction<br>Sex: Female<br>Inclusion: confirmed invasive/non-invasive primary breast cancer schedule for surgical excision, at high risk of shoulder problems as defined by criteria given in Table 2.<br>Exclusion: males, and women with exclusion criteria as described in Table 2.
Study type	Interventional<br>Allocation: randomised; individual assignment.<br>Primary purpose: prevention.<br>Phase III
Date of first enrolment	January 2016
Target sample size	350
Recruitment status	Recruiting to July 2017
Primary outcome	Arm, shoulder and hand function as measured using the Disabilities of the Arm, Shoulder and Hand (DASH) questionnaire at 12 months
Key secondary outcomes	DASH subscales, pain (acute, chronic, neuropathic), health-related quality of life, surgical site infection, lymphoedema and other complications, health care resource use.

For peer review only - http://bmjopen.bmj.com/site/about/guidelines.xhtml

	Exercise/activity data to inform adherence to interventions.

For peer review only

For peer review only - http://bmjopen.bmj.com/site/about/guidelines.xhtml

Table 2. Trial inclusion and exclusion criteria

**Inclusion Criteria**	**Exclusion Criteria**
Aged ≥18 years old	Males
Histologically confirmed invasive or non-invasive primary breast cancer scheduled for surgical excision of breast cancer	Women having immediate reconstructive surgery
Considered high risk of developing shoulder problems after surgery defined by one or more of the following:<br>• Planned axillary node clearance<br>• Planned radiotherapy to the axilla and/or supraclavicular*<br>• Existing shoulder problems (based upon PROSPER screening criteria)	Women having sentinel lymph node biopsy (SLNB), with or without breast surgery, unless they fulfil other high risk criteria
Obesity defined as BMI >30	Women having bilateral breast surgery
Any subsequent axillary surgery related to primary surgery e.g. ANC conducted after sentinel lymph node biopsy (SLNB)	Evidence of metastatic disease at time of recruitment
Able to provide written informed consent	
Willing and able to comply with the protocol	
**includes women informed of need for radiotherapy to the axilla and/or supraclavicular within six weeks of surgery, thus potential late entry to the trial is allowed in this setting*	

For peer review only - http://bmjopen.bmj.com/site/about/guidelines.xhtml

Figure 1. Study outcome measures and assessment time points

	Study period				
	**Enrolment**	**Allocation**	**Post-allocation**		
**Time point**	$-t_1$	*0*	t_1 6 weeks	t_2 6 months	t_3 12 months
Enrolment:					
Eligibility screen	√				
Informed consent	√				
Randomisation	350	√			
Interventions					
Usual Care (UC)	175	All participants	←→		
UC + PROSPER intervention	175		←	—	→
Assessments:					
Baseline	√				
Primary outcome	Function	DASH			√
Secondary outcomes	Function	DASH subscales	-	√	√
	Acute & chronic pain	FACT-B+4; NRS	√	√	√
	Neuropathic pain	DN4	√	√	√
	Complications	SSI + self-report	√	√	√
	Lymphoedema	Self-report	√	√	√
	Health-related QoL	SF12 / EQ-5D-5L	-	√	√
	Resource use	Self-report	-	√	√
	General activity & exercise	PASE items	√	√	√

DASH: Disabilities of Arm, Shoulder and Hand questionnaire; DN4: Doleur Neuropathique; SF12: Short Form-12; SSI: surgical site infection; EQ-5D-5L: Euroqol; t: time point; QoL: Quality of Life; PASE: Physical Activity Scale for the Elderly.

For peer review only

For peer review only - http://bmjopen.bmj.com/site/about/guidelines.xhtml

Figure 2. Trial flow diagram

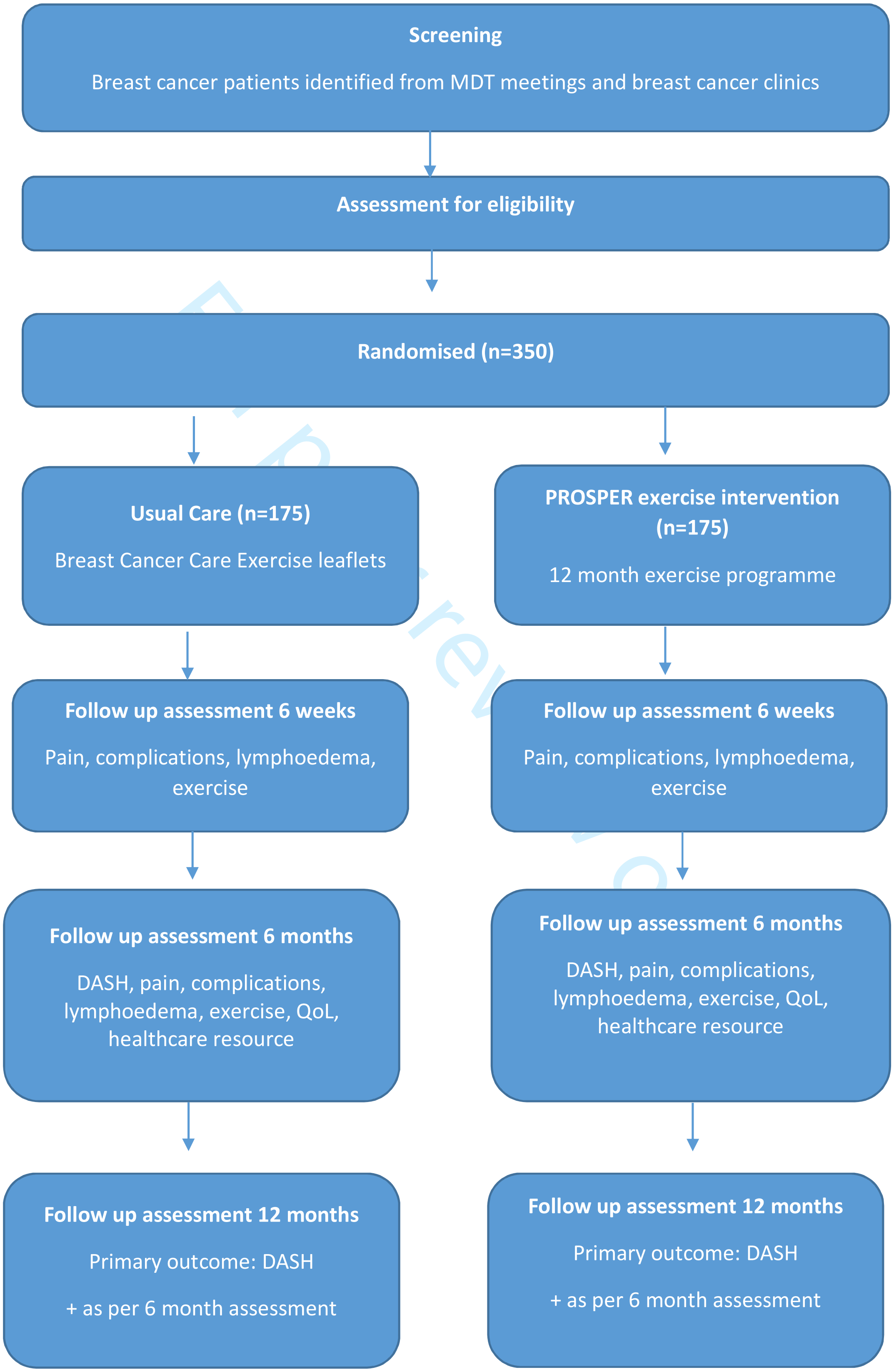

For peer review only - http://bmjopen.bmj.com/site/about/guidelines.xhtml

SPIRIT

STANDARD PROTOCOL ITEMS: RECOMMENDATIONS FOR INTERVENTIONAL TRIALS

SPIRIT 2013 Checklist: Recommended items to address in a clinical trial protocol and related documents*

Section/item	Item No	Description	Addressed on page number
**Administrative information**			
Title	1	Descriptive title identifying the study design, population, interventions, and, if applicable, trial acronym	1
Trial registration	2a	Trial identifier and registry name. If not yet registered, name of intended registry	4
	2b	All items from the World Health Organization Trial Registration Data Set	Table 1
Protocol version	3	Date and version identifier	3 & 22
Funding	4	Sources and types of financial, material, and other support	25 & Table 1
Roles and responsibilities	5a	Names, affiliations, and roles of protocol contributors	24-25
	5b	Name and contact information for the trial sponsor	Table 1
	5c	Role of study sponsor and funders, if any, in study design; collection, management, analysis, and interpretation of data; writing of the report; and the decision to submit the report for publication, including whether they will have ultimate authority over any of these activities	25-6
	5d	Composition, roles, and responsibilities of the coordinating centre, steering committee, endpoint adjudication committee, data management team, and other individuals or groups overseeing the trial, if applicable (see Item 21a for data monitoring committee)	24

For peer review only - http://bmjopen.bmj.com/site/about/guidelines.xhtml

For peer review only

**Introduction**			
Background and rationale	6a	Description of research question and justification for undertaking the trial, including summary of relevant studies (published and unpublished) examining benefits and harms for each intervention	5 - 7
	6b	Explanation for choice of comparators	10 – 12
Objectives	7	Specific objectives or hypotheses	8
Trial design	8	Description of trial design including type of trial (eg, parallel group, crossover, factorial, single group), allocation ratio, and framework (eg, superiority, equivalence, noninferiority, exploratory)	8 - 9
**Methods: Participants, interventions, and outcomes**			
Study setting	9	Description of study settings (eg, community clinic, academic hospital) and list of countries where data will be collected. Reference to where list of study sites can be obtained	8
Eligibility criteria	10	Inclusion and exclusion criteria for participants. If applicable, eligibility criteria for study centres and individuals who will perform the interventions (eg, surgeons, psychotherapists)	8 – 9
Interventions	11a	Interventions for each group with sufficient detail to allow replication, including how and when they will be administered	10-12 + separate intervention papers
	11b	Criteria for discontinuing or modifying allocated interventions for a given trial participant (eg, drug dose change in response to harms, participant request, or improving/worsening disease)	22
	11c	Strategies to improve adherence to intervention protocols, and any procedures for monitoring adherence (eg, drug tablet return, laboratory tests)	12
	11d	Relevant concomitant care and interventions that are permitted or prohibited during the trial	12
Outcomes	12	Primary, secondary, and other outcomes, including the specific measurement variable (eg, systolic blood pressure), analysis metric (eg, change from baseline, final value, time to event), method of aggregation (eg, median, proportion), and time point for each outcome. Explanation of the clinical relevance of chosen efficacy and harm outcomes is strongly recommended	Table 2
Participant timeline	13	Time schedule of enrolment, interventions (including any run-ins and washouts), assessments, and visits for participants. A schematic diagram is highly recommended (see Figure)	Figure 1

For peer review only - http://bmjopen.bmj.com/site/about/guidelines.xhtml

Sample size	14	Estimated number of participants needed to achieve study objectives and how it was determined, including clinical and statistical assumptions supporting any sample size calculations	16
Recruitment	15	Strategies for achieving adequate participant enrolment to reach target sample size	9
**Methods: Assignment of interventions (for controlled trials)**			
Allocation:			
Sequence generation	16a	Method of generating the allocation sequence (eg, computer-generated random numbers), and list of any factors for stratification. To reduce predictability of a random sequence, details of any planned restriction (eg, blocking) should be provided in a separate document that is unavailable to those who enrol participants or assign interventions	9
Allocation concealment mechanism	16b	Mechanism of implementing the allocation sequence (eg, central telephone; sequentially numbered, opaque, sealed envelopes), describing any steps to conceal the sequence until interventions are assigned	9
Implementation	16c	Who will generate the allocation sequence, who will enrol participants, and who will assign participants to interventions	10
Blinding (masking)	17a	Who will be blinded after assignment to interventions (eg, trial participants, care providers, outcome assessors, data analysts), and how	10
	17b	If blinded, circumstances under which unblinding is permissible, and procedure for revealing a participant's allocated intervention during the trial	10
**Methods: Data collection, management, and analysis**			
Data collection methods	18a	Plans for assessment and collection of outcome, baseline, and other trial data, including any related processes to promote data quality (eg, duplicate measurements, training of assessors) and a description of study instruments (eg, questionnaires, laboratory tests) along with their reliability and validity, if known. Reference to where data collection forms can be found, if not in the protocol	11 & Table 2
	18b	Plans to promote participant retention and complete follow-up, including list of any outcome data to be collected for participants who discontinue or deviate from intervention protocols	11 & 14

For peer review only - http://bmjopen.bmj.com/site/about/guidelines.xhtml

Data management	19	Plans for data entry, coding, security, and storage, including any related processes to promote data quality (eg, double data entry; range checks for data values). Reference to where details of data management procedures can be found, if not in the protocol	20
Statistical methods	20a	Statistical methods for analysing primary and secondary outcomes. Reference to where other details of the statistical analysis plan can be found, if not in the protocol	17-20
	20b	Methods for any additional analyses (eg, subgroup and adjusted analyses)	17-20
	20c	Definition of analysis population relating to protocol non-adherence (eg, as randomised analysis), and any statistical methods to handle missing data (eg, multiple imputation)	18-20
**Methods: Monitoring**			
Data monitoring	21a	Composition of data monitoring committee (DMC); summary of its role and reporting structure; statement of whether it is independent from the sponsor and competing interests; and reference to where further details about its charter can be found, if not in the protocol. Alternatively, an explanation of why a DMC is not needed	22
	21b	Description of any interim analyses and stopping guidelines, including who will have access to these interim results and make the final decision to terminate the trial	20
Harms	22	Plans for collecting, assessing, reporting, and managing solicited and spontaneously reported adverse events and other unintended effects of trial interventions or trial conduct	22
Auditing	23	Frequency and procedures for auditing trial conduct, if any, and whether the process will be independent from investigators and the sponsor	12 & described in intervention papers
**Ethics and dissemination**			
Research ethics approval	24	Plans for seeking research ethics committee/institutional review board (REC/IRB) approval	22
Protocol amendments	25	Plans for communicating important protocol modifications (eg, changes to eligibility criteria, outcomes, analyses) to relevant parties (eg, investigators, REC/IRBs, trial participants, trial registries, journals, regulators)	4

For peer review only - http://bmjopen.bmj.com/site/about/guidelines.xhtml

Consent or assent	26a	Who will obtain informed consent or assent from potential trial participants or authorised surrogates, and how (see Item 32)	9
	26b	Additional consent provisions for collection and use of participant data and biological specimens in ancillary studies, if applicable	9 & 21
Confidentiality	27	How personal information about potential and enrolled participants will be collected, shared, and maintained in order to protect confidentiality before, during, and after the trial	9
Declaration of interests	28	Financial and other competing interests for principal investigators for the overall trial and each study site	24
Access to data	29	Statement of who will have access to the final trial dataset, and disclosure of contractual agreements that limit such access for investigators	26
Ancillary and post-trial care	30	Provisions, if any, for ancillary and post-trial care, and for compensation to those who suffer harm from trial participation	12
Dissemination policy	31a	Plans for investigators and sponsor to communicate trial results to participants, healthcare professionals, the public, and other relevant groups (eg, via publication, reporting in results databases, or other data sharing arrangements), including any publication restrictions	3-4 & 20
	31b	Authorship eligibility guidelines and any intended use of professional writers	3
	31c	Plans, if any, for granting public access to the full protocol, participant-level dataset, and statistical code	24
**Appendices**			
Informed consent materials	32	Model consent form and other related documentation given to participants and authorised surrogates	Can be provided as Appendix if requested
Biological specimens	33	Plans for collection, laboratory evaluation, and storage of biological specimens for genetic or molecular analysis in the current trial and for future use in ancillary studies, if applicable	Not applicable

*It is strongly recommended that this checklist be read in conjunction with the SPIRIT 2013 Explanation & Elaboration for important clarification on the items. Amendments to the protocol should be tracked and dated. The SPIRIT checklist is copyrighted by the SPIRIT Group under the Creative Commons "Attribution-NonCommercial-NoDerivs 3.0 Unported" license.

For peer review only - http://bmjopen.bmj.com/site/about/guidelines.xhtml

BMJ Open

A randomised controlled trial of exercise to prevent shoulder problems in women undergoing breast cancer treatment: study protocol for the Prevention of Shoulder Problems Trial (UK PROSPER Trial)

Journal:	*BMJ Open*
Manuscript ID	bmjopen-2017-019078.R1
Article Type:	Protocol
Date Submitted by the Author:	01-Nov-2017
Complete List of Authors:	Bruce, Julie; University of Warwick, Warwick Clinical Trials Unit<br>Williamson, Esther; Nuffield Department of Orthopaedics, Rheumatology and Musculoskeletal Sciences, University of Oxford, United Kingdom. , Centre for Rehabilitation Research in Oxford<br>Lait, Clare; Gloucestershire Care Services NHS Trust<br>Richmond, Helen ; University of Warwick, Warwick Clinical Trials Unit<br>Betteley, Lauren; University of Warwick Warwick Medical School, Warwick Clinical Trials Unit<br>Lall, Ranjit; University of Warwick, Warwick Clinical Trials Unit<br>Petrou, Stavros; University of Warwick, Warwick Medical School<br>Rees, Sophie; University of Warwick, Warwick Clinical Trials Unit<br>Withers, Emma; University of Warwick, Warwick Clinical Trials Unit<br>Lamb, Sarah; University of Oxford, Nuffield Department of Orthopaedics, Rheumatology and Musculoskeletal Sciences<br>Thompson, Alastair; University of Texas M.D. Anderson Cancer Center, Department of Breast Surgical Oncology and Department of Translational Molecular Pathology
**Primary Subject Heading**:	Rehabilitation medicine
Secondary Subject Heading:	Surgery, Health services research, Oncology
Keywords:	Breast surgery < SURGERY, Clinical trials < THERAPEUTICS, Rehabilitation medicine < INTERNAL MEDICINE

SCHOLARONE™
Manuscripts

A randomised controlled trial of exercise to prevent shoulder problems in women undergoing breast cancer treatment: study protocol for the Prevention of Shoulder Problems Trial (UK PROSPER Trial)

Authors: Julie Bruce[1], Esther Williamson[2], Clare Lait[3], Helen Richmond[1], Lauren Betteley[1], Ranjit Lall[1], Stavros Petrou[1], Sophie Rees[1], Emma J Withers[1], Sarah E Lamb[1,2] and Alastair Thompson[4] on behalf of the PROSPER Study Group.

Author affiliations

1. Warwick Clinical Trials Unit, Division of Health Sciences, University of Warwick, Coventry CV4 7AL, UK
2. Nuffield Department of Orthopaedics Rheumatology & Musculoskeletal Sciences, University of Oxford, Windmill Road, Oxford, OX3 7LD, UK
3. Gloucestershire Care Services NHS Trust, 1010 Gloucester Business Park, Pioneer Avenue, Brockworth, Gloucester, GL3 4AW, UK
4. Department of Breast Surgical Oncology & Department of Translational Molecular Pathology, University of Texas M.D. Anderson Cancer Center, 1400 Pressler Drive, Houston, Texas, US

Corresponding author: *Professor Julie Bruce; julie.bruce@warwick.ac.uk*

For peer review only

For peer review only - http://bmjopen.bmj.com/site/about/guidelines.xhtml

Abstract

Musculoskeletal shoulder problems are common after breast cancer treatment. Early postoperative exercises targeting the upper limb may improve shoulder function. This protocol describes a National Institute for Health Research (NIHR) funded randomised controlled trial (RCT) to evaluate the clinical and cost-effectiveness of an early supervised structured exercise programme compared to usual care, for women at high risk of developing shoulder problems after breast cancer surgery.

Methods

This pragmatic two-armed, multicentre RCT is underway within secondary care in the United Kingdom (UK). PROSPER aims to recruit 350 women from approximately 15 UK centres with follow-up at 6 weeks, 6 and 12 months after randomisation. Recruitment processes and intervention development were optimised through qualitative research during a six-month internal pilot phase. Participants are randomised to the PROSPER intervention or best practice usual care only. The PROSPER intervention is delivered by physiotherapists and incorporates three main components: shoulder-specific exercises targeting range of movement and strength; general physical activity; and behavioural strategies to encourage adherence and support exercise behaviour. The primary outcome is upper arm function assessed using the Disabilities of the Arm Shoulder and Hand (DASH) questionnaire at 12 months post-randomisation. Secondary outcomes include DASH subscales, acute and chronic pain, complications, health related quality of life, and healthcare resource use. We will interview a subsample of twenty participants to explore their experiences of the trial interventions.

Discussion

The PROSPER study is the first multicentre UK clinical trial to investigate the clinical and cost-effectiveness of supported exercise in the prevention of shoulder problems in high risk women undergoing breast cancer surgery. The findings will inform future clinical practice and provide valuable insight into the role of physiotherapy-supported exercise in breast cancer rehabilitation.

For peer review only - http://bmjopen.bmj.com/site/about/guidelines.xhtml

For peer review only

Trial registration: ISRCTN35358984

Protocol version: Version 2.1; dated 11/01/2017

Funding: NIHR HTA (Project Number 13/84/10)

Strengths and limitations of this study

Strengths:

- A large pragmatic study delivering a complex intervention to prevent postoperative health problems in newly diagnosed cancer patients within secondary care;
- A strength of the evaluation is the mixed methods approach incorporating embedded qualitative research and economic analysis

Limitations:

- Recruited participants undergo multiple cancer treatments thus experience a complicated postoperative recovery pathway.

For peer review only

For peer review only - http://bmjopen.bmj.com/site/about/guidelines.xhtml

Background

Breast cancer is the most common cancer in women in the UK with a 30% increase in incidence since the early 1970s (1). Due to advances in screening and treatment, the survival rate has progressively risen and now two thirds of women will survive for 20 years beyond their breast cancer diagnosis (1). The mainstay treatment is surgery to the breast and axilla, supplemented with chemotherapy, radiotherapy and endocrine therapy, depending upon tumour stage and other clinical criteria. As a consequence of these treatments, upper limb problems such as decreased shoulder range of movement (ROM), impaired strength, chronic pain, sensory disturbances and lymphoedema are common adverse treatment effects (2, 3). Studies suggest that up to 67% of women have arm or shoulder symptoms up to 3 years after treatment (4). Persistent upper limb dysfunction and pain are debilitating, have a negative impact upon sleep, quality of life, physical functioning and emotional wellbeing. Given successes in increasing survival, it is timely to identify strategies to improve the health-related quality of life of women after breast cancer treatment.

Several risk factors for shoulder and upper body problems after breast cancer treatment have been identified, including treatment-related factors, such as type of axillary surgery and radiotherapy (RT), and patient factors such as age, body mass index (BMI), and pre-existing shoulder problems (4, 5). Women undergoing mastectomy compared to breast conserving surgery are at greater risk of postoperative shoulder restrictions (odds ratio (OR) 5.7, 95% confidence interval (CI) 1.03, 31.2) (4). Additionally, those undergoing axillary lymph node clearance (ANC) are at greater risk of postoperative arm complaints compared to those having sentinel lymph node biopsy (SLNB; OR 9.8; 95% CI 3.5, 27.5) (4-6). Radiotherapy (RT), particularly to the axilla or chest wall, increases the odds of shoulder restriction (pooled OR 1.7, 95% CI 1.0, 2.9) and lymphoedema (pooled OR 1.5, 95% CI 1.2, 1.8) compared to women treated without adjuvant RT (4). Women reporting problems in the upper body and shoulder region before surgery are also at increased risk of chronic postoperative pain (5, 7).

For peer review only - http://bmjopen.bmj.com/site/about/guidelines.xhtml

BMI at time of surgery has been shown to have an independent negative effect on shoulder external rotation up to seven years after breast cancer treatment, and increased BMI is a risk factor for chronic postoperative pain and arm lymphoedema (7, 8). It is important that the UK National Health Service (NHS) provides optimal care for these women at high risk of developing shoulder problems to ensure recovery and return to usual activities after cancer treatment.

A Cochrane review identified 24 studies (2132 participants) investigating exercise following breast cancer surgery (9). Six studies (n=354), conducted outside of the UK, found that structured postoperative exercise significantly improved shoulder ROM in the short and long term when compared to usual care (9). Ten studies (n=1304) have evaluated timing of exercise delivery; programmes initiated immediately postoperatively (1-3 days) versus delayed exercise suggest that early postoperative exercise does significantly improve long-term shoulder ROM. However, some studies reported an increased risk of wound-related complications with early exercise, such as seroma and surgical site infection (9). The largest UK trial to date (n=116 patients), published after the Cochrane review, found that participants were less likely to develop lymphoedema when exercises were limited to 90° of shoulder elevation during the first postoperative week compared to those performing unrestricted exercises (10). These previous trials investigating the efficacy of exercise following breast cancer surgery have been criticised for being of poor methodological quality and for omitting important patient-reported outcomes such as function and health-related quality of life (9). Furthermore, there is ongoing uncertainty around the optimal type, dose, and timing of exercise after breast cancer treatment. Moreover, none of the trials conducted to date have investigated the cost-effectiveness of structured exercise programmes after breast cancer treatment.

For peer review only - http://bmjopen.bmj.com/site/about/guidelines.xhtml

Rationale for a trial

To date, no large scale, high-quality, multicentre RCT investigating the clinical effectiveness of a structured physiotherapy intervention compared to usual care for women undergoing breast cancer surgery has been conducted. Given the lack of knowledge regarding the intensity and duration of exercise interventions after breast cancer surgery, this trial will provide evidence on whether a rigorously designed physiotherapy-led intervention, incorporating behaviour change theory, improves postoperative function and related outcomes, and whether this is cost-effective to deliver in the NHS setting.

Methods

Aim

The overall aim of PROSPER is to investigate the clinical and cost-effectiveness of an early supervised exercise programme compared to best practice usual care for women at high risk of shoulder problems after treatment for breast cancer, on outcomes of upper arm function, complications and quality of life. Specific trial objectives are:

1. To develop and refine a complex intervention of physiotherapy-led exercise for women at risk of developing musculoskeletal problems after breast cancer treatment;
2. To assess the acceptability of the structured exercise programme and outcome measures, optimise participant recruitment and refine trial processes during a six-month internal pilot phase at three clinical centres;
3. Use findings from the internal pilot phase to undertake a definitive full RCT in approximately 15 UK NHS breast cancer centres.

This protocol follows guidance from the Standard Protocol Items: Recommendations for Interventional Trials (SPIRIT)(11). Core trial information is presented in Table 1 (WHO Trial Registration Data Set). Figure 1 details the schedule of enrolment, interventions and assessment.

For peer review only - http://bmjopen.bmj.com/site/about/guidelines.xhtml

Trial design and setting

A multicentre, pragmatic, parallel, two-arm RCT with an internal pilot study and embedded economic evaluation and qualitative studies. The trial framework is superiority rather than equivalence or exploratory. The trial is currently open and recruiting from 17 NHS tertiary breast cancer centres across England. Participants are randomised in a 1:1 ratio between intervention and control arms.

Patient and public involvement (PPI) in trial design

Four female PPI representatives, all of whom were treated for breast cancer, were consulted during the initial grant preparation, intervention development and trial set up. Our PPI representatives contributed to the design of the intervention and advised on recruitment-related issues; they provided valuable insight into the worries and concerns experienced during cancer treatment.

Eligibility Criteria

Women are eligible to participate in PROSPER if they are: diagnosed with histologically confirmed invasive or non-invasive primary breast cancer scheduled for surgical excision; aged 18 years or over; can comply with the protocol; willing to provide written informed consent; and considered as being at high risk of developing postoperative shoulder problems (Table 2). This is a pragmatic trial and it is important that inclusion criteria reflect contemporary clinical practice. Therefore, women are also eligible where a later decision is made for postoperative radiotherapy (RT) to the axilla and/or supraclavicular region, thus changing their risk status from low to high. 'Late entry' women are eligible for the trial if the decision for postoperative RT is made within six weeks of surgery. Women who have had previous breast surgery (such as excision of a benign tumour or breast cyst) and those women who have had previous contralateral (opposite side) mastectomy, are eligible for invitation providing they fulfil high risk criteria for shoulder problems. Women having

For peer review only - http://bmjopen.bmj.com/site/about/guidelines.xhtml

immediate reconstruction or bilateral breast surgery are ineligible as the usual NHS postoperative care pathway often includes routine postoperative physiotherapy. Exclusion criteria are presented in Table 2.

Participant screening, recruitment and consent

Participants are screened and identified from multi-disciplinary team (MDT) meetings and preoperative breast/oncology clinic lists in secondary care. The initial screening process is undertaken by a member of the clinical team, research nurse or trained designee. Potentially eligible patients are approached by clinical or research staff and are given a Patient Information Sheet (PIS) with further explanation of the trial. Figure 2 summarises participant flow.

Allocation sequence generation and randomisation

Randomisation is based upon a computer-generated algorithm held and controlled centrally by the Warwick Clinical Trials Unit (WCTU) programming team, independent from the PROSPER trial team. The WCTU telephone randomisation service is used whereby randomisation occurs after eligibility and informed consent has been obtained. Concealment of allocation is maintained. An automated confirmation email of intervention allocation is generated to the study team. Randomisation is stratified by the following variables: (i) first versus repeat surgery; (ii) centre; and (iii) whether informed of the need for radiotherapy within six weeks of surgery. The first variable adjusts for the requirement for any additional surgery which may change risk status from low to high (e.g. second procedure ANC or reexcision of surgical margins). The second stratification variable ensures balanced allocation across each recruitment site. The third variable accounts for late entry to the trial, thus relates to the timing of intervention delivery and whether participants are randomised preoperatively (up to the day of surgery) or within the first six weeks postoperatively. Due to the nature of the study intervention, it is not possible to blind participants or treating physiotherapists to treatment allocation. However, receipt and handling of outcome data

For peer review only - http://bmjopen.bmj.com/site/about/guidelines.xhtml

collection is blinded, thus data entry of returned postal questionnaires, data cleaning and interim statistical analyses are conducted without knowledge of treatment allocation (blinded).

Interventions

Control Arm: Usual Care

All participants allocated to the usual care arm receive best practice usual care in the form of written leaflets containing information about exercises, recovery after surgery, and treatments for breast cancer. During the pilot phase, different exercise information leaflets were reviewed and considered; we also consulted best practice guidance for written patient information materials (12). The most commonly used information leaflets were 'Exercises after Breast Cancer Surgery (BCC6)' and 'Your Operation and Recovery (BCC151)' published by Breast Cancer Care (BCC) (13). The BCC leaflets were selected because of content, style and clarity of presentation of information. These two information leaflets were given to all patients before surgery by breast care nurses, or other healthcare professionals, depending upon local practice.

Intervention Arm: PROSPER exercise programme

Participants randomised to the active intervention receive usual care leaflets in addition to the PROSPER intervention: a structured individualised exercise programme, comprising a minimum of three face-to-face and maximum of six sessions or contacts with a physiotherapist. A more detailed description of intervention development and final content has been submitted for publication. In brief, the PROSPER programme comprises specific exercises targeting shoulder range of motion and upper arm muscle strength, general physical activity, and behavioural adherence strategies. The intervention is predominantly delivered in physiotherapy outpatient departments.

For peer review only - http://bmjopen.bmj.com/site/about/guidelines.xhtml

The first physiotherapy session is arranged seven to ten days after surgery, for assessment of shoulder ROM, postoperative pain, function, arm swelling, patients' goals and assessment of confidence to carry out prescribed exercises. Participants are prescribed an individually tailored home exercise programme and provided with guidance on rehabilitation, management of postoperative complications and returning to general physical activity and/or work. The second appointment is between four to six weeks postoperatively to review progress and prescribe shoulder strengthening exercises. The programme is progressed by increasing exercise repetitions, sets and resistance. The third appointment is recommended for between 12 to 16 weeks postoperatively, for further progression to facilitate return to work, sport and hobbies. For women with later entry on the basis of postoperative radiotherapy, these timings will be slightly delayed, but the exercise programme should commence at the earliest opportunity, thus within six weeks of surgery.

As per development work with patient representatives, and to reflect the pragmatic trial design, three additional physiotherapy consultations are available on request. The timing and delivery of additional appointments, either via telephone or face-to-face, are flexible to account for on-going treatment, physiotherapist judgement and patient preference. Ideally the intervention will be completed within the first six months following surgery, but women can contact their physiotherapist for up to 12 months after randomisation. Thus any late treatment-related shoulder problems will be dealt with by the trial physiotherapist. Number and method of physiotherapy contacts will be closely monitored during the trial.

Outcomes

Figure 1 and Table 3 present the study outcome measures and standardised assessment scales by assessment time point. Questionnaires are completed at baseline on recruitment, then at 6 weeks, 6 and 12 months after randomisation by post. The **primary outcome** is upper limb function at 12 months measured using the Disabilities of Arm, Shoulder and Hand (DASH) questionnaire. We considered other patient-reported outcome measures, including

For peer review only - http://bmjopen.bmj.com/site/about/guidelines.xhtml

shoulder-specific scales, however selected the DASH because it captures symptoms and function of the upper limb rather than the shoulder joint *per se.* There is good evidence to suggest that women experience a variety of difficulties and restrictions after breast cancer treatment, affecting the hand, arm and shoulder. Functional impairment to the arm can affect performance of simple daily activities, including writing, opening or closing jars, lifting and/or holding shopping bags.

The DASH is a 30-item patient-reported outcome measure designed to capture difficulty in performing various upper arm activities (14). A single DASH score is generated, although psychometric assessment using discriminant content validation analysis has shown that the scale can be used to produce three health outcome sub-scores for impairment, activity limitation and participation restriction, as per the WHO International Classification of Functioning Disability and Health (ICF) taxonomy (15).

Secondary outcomes include health-related quality of life (EuroQol EQ-5D-5L and Short-Form-12), DASH sub-scores, and surgical adverse events including pain (acute, chronic, neuropathic pain) surgical site infection and lymphoedema. A numerical rating scale (NRS) 0-10 and Doleur Neuropathique Questionnaire (DN4) are used to collect pain intensity and pain character. The Functional Assessment of Cancer Therapy-Breast (FACT-B4) subscale captures arm tenderness, numbness, painful movement and stiffness. We added items to capture arm heaviness and swelling as self-report indicators of lymphoedema.

Data on exercise/mobility are collected to allow comparisons in physical activity (selected items from the Physical Activity Scale for the Elderly (PASE)). Healthcare resource use is recorded for economic analyses.

Sample Size

The PROSPER trial aims to recruit 350 patients, allocated in a 1:1 ratio. The sample size calculation is based on a Dutch trial of thirty women with breast cancer, randomised to

For peer review only - http://bmjopen.bmj.com/site/about/guidelines.xhtml

physiotherapy over a three month period, reporting a between group difference of 7 points on the DASH at 6 months (16). At 80% power and p<0.05, this yields a target of 242 participants in total. Accounting for therapist effects, an intracluster coefficient (ICC) of 0.01 (yielding a design effect of 1.05), gives a target of 256 participants. The ICC estimate is based on our previous experience of exercise interventions in a range of musculoskeletal trials. We anticipate loss to follow up of less than 10% based on our previous clinical trials however, have inflated this to 25% to cover the possibility that numbers lost to follow up are greater than anticipated e.g. due to ongoing cancer treatment.

The study is powered to detect a 7 point difference on the DASH. Studies of rheumatological and orthopaedic populations have suggested that the minimally clinically important difference (MCID) for the DASH is 10, and that the between group difference for trials should be set at 10 (17). However, this fails to account for many of the eventualities that occur in pragmatic trials, notably that there is not a 'no treatment' control arm, and therefore that some of the control group may be exposed by serendipity to an intervention of similar intensity, particularly in a high risk population.

Internal pilot study

A six-month internal pilot phase was conducted at three breast cancer units (Coventry, Oxford and Wolverhampton) to evaluate processes for patient identification, eligibility and refinement of recruitment estimates. The intended sample size for the internal pilot study was 30 participants, approximately 10% of the full sample. Acceptability of the PROSPER intervention was explored through qualitative research involving audio-recorded individual interviews with seven participants. Changes were made to patient-facing materials and to exercise intervention materials. Easy to use pocket-sized laminated cards with details of inclusion/exclusion criteria and shoulder screening criteria were produced for recruitment staff. Additional telephone or face-to-face appointments were added to the exercise intervention to allow for flexibility during ongoing cancer treatment. Data from the pilot phase

For peer review only - http://bmjopen.bmj.com/site/about/guidelines.xhtml

helped to refine recruitment and trial processes. Patients recruited to the pilot phase continue with the follow-up schedule and will be retained in the full trial analysis. The pilot study was completed as planned and the funder approved progression to full trial.

Data analysis

Statistical analysis will be intention-to-treat and will comply with the CONSORT guidelines. The primary outcome data will be summarised using mean, standard deviation, median and range values. The clustering effect will be assessed prior to analysis of the data. In the presence of a clustering effect, the primary outcome will be analysed using multi-level linear regression models. If there is negligible clustering effect, it will be analysed using ordinary linear regression models. In each case, the mean change from baseline (to 6 and 12 months) will be summarised for each of the treatment arms and differences between the interventions using unadjusted and adjusted (for age, type of surgery and radiotherapy) estimates. These mean changes and their 95% confidence intervals will be plotted graphically so that change can be assessed over the course of the study. Continuous secondary outcomes will be assessed in a similar way to the primary outcome. Categorical data will be analysed using random effect/ordinary logistic models, depending on the presence of a clustering effect.

A DASH score cannot be computed if there are more than three missing items. As a sensitivity analysis, the impact of missing data will be assessed using multiple imputation. The impact of non-compliance with the intervention will be examined using the complier average causal effect (CACE) analysis (18, 19). We have reviewed definitions of compliance for CACE analyses used in other therapy trials (20, 21). Complete compliance with the PROSPER intervention is defined as having three or more contacts with the PROSPER therapist; an additional analysis will be undertaken to explore partial compliance, defined as less than three sessions. Analyses and template tables will be reported in a detailed statistical analysis plan for review and approval by the DMC, prior to final statistical analysis

For peer review only - http://bmjopen.bmj.com/site/about/guidelines.xhtml

of the data. Planned sensitivity analyses include: a) the impact of low/high recruitment centres on clustering effect; and b) assessment of differences between date of randomisation and date of surgery across groups, as surgical trials vary in relation to timing of follow-up.

Economic Evaluation

The primary economic evaluation will be conducted from the NHS and personal social services (PSS) perspective (22) using the intention-to-treat approach (23). Data will be collected on the health and social service resources used in the treatment of each trial participant from randomisation to 12 months post-randomisation. Primary research methods will be used to estimate the costs of delivering the physiotherapy-led exercise programme, including development and training of accredited providers, the cost of delivering the individual sessions and participant monitoring activities. Broader resource utilisation will be captured through three main sources: (i) clinical data extraction forms; (ii) patient postal questionnaires at 6 months and 12 months post-randomisation; and if feasible within the trial timeline, (iii) routine health data sources from NHS Digital. Current UK unit costs, will be applied to each resource item to estimate costs in each trial arm. Health-related quality of life will be measured at baseline and at 6 and 12 months post-randomisation using the generic EQ-5D-5L and SF-12 measures; national tariff sets will be used to generate quality-adjusted life-years (QALYs) (24-28).

An incremental cost-effectiveness analysis, expressed in terms of incremental cost per QALY gained, will be performed. Detailed methods of analysis will be pre-specified within a health economics analysis plan approved by the trial team prior to analysis to ensure appropriate methods are used. Results will be presented using incremental cost-effectiveness ratios (ICERs) and cost-effectiveness acceptability curves generated via the net-benefit framework. A series of sensitivity analyses will be undertaken to explore the implications of uncertainty on the ICERs and to consider the broader issue of the

For peer review only - http://bmjopen.bmj.com/site/about/guidelines.xhtml

generalisability of the study results. Due to the known limitations of within-trial economic evaluations (29), a decision-analytical model may be constructed to examine the longer term costs and outcomes beyond the end of the trial. Costs and outcomes beyond the first year will be discounted to present values (22) and probabilistic sensitivity analyses will be undertaken to explore the impact of uncertainty on the ICERs.

Qualitative sub-study

An embedded qualitative study will be undertaken to gain insight into the experiences of women participating in trial interventions. We will explore the acceptability of the exercise programme and compare and contrast experiences with women allocated to the control intervention.

Design of sub-study

In-depth, semi-structured interviews will be conducted and audio-recorded. Interview topic guides will be used to ensure similar areas are covered in each interview. Participants consenting to the main trial are asked to indicate willingness to take part in a future interview to explore postoperative experiences. A total of twenty interviews are planned, with ten women from each intervention arm. Purposive sampling will be used, striving for a mix of geographical location, age, employment status, socio-economic background and ethnicity.

Analysis

Interviews will be recorded, transcribed and analysed using a Framework Approach. A thematic framework will be developed using pre-determined themes plus new themes raised by participants. The framework will be applied to the interview text and coded data will be arranged on a chart according to each theme identified. Themes will be examined with a view to providing explanations of the participants' experiences and understandings.

For peer review only - http://bmjopen.bmj.com/site/about/guidelines.xhtml

Data security and management

Participant data is stored on a secure database in accordance with the Data Protection Act (1998). A unique trial identification number is used on all participant communication. Clinical and patient forms are being checked for completeness and congruity before data entry onto the PROSPER trial database. Data will undergo additional checks to ensure consistency between data submitted and original paper forms. Trial documentation and data will be archived for at least ten years after completion of the trial in accordance with WCTU standard operating procedures.

Trial monitoring

The Trial Management Group (TMG) will oversee all aspects of design, delivery, quality assurance and data analysis. A Trial Steering Committee (TSC), with independent Chairperson, will monitor the trial at least once per year. An independent DMC will review trial progress, recruitment, protocol compliance and interim analysis of outcomes, annually or more frequently as requested. Recruitment data from the internal pilot study were reviewed by independent committees and by the funder to approve the launch of the main trial.

Adverse event management

A safety reporting protocol has been developed for related and unexpected serious adverse events (SAEs) and directly attributable adverse events (AEs). An AE is defined as any untoward medical occurrence in a subject which does not necessarily have a causal relationship with the intervention. Any adverse event that occurs whilst undertaking PROSPER exercises, either during an appointment, or whilst exercising unsupervised at home, require reporting to the trial team. The trial Chief Investigator, with input from the WCTU Quality Assurance team, determine whether AEs require reporting to the trial sponsor, DMC and Ethics Committee, in accordance with the full safety reporting protocol.

For peer review only - http://bmjopen.bmj.com/site/about/guidelines.xhtml

Research ethics approval

Ethical approval was granted from the NHS National Research Ethics Service (NRES) Committee West Midlands (Solihull) (15/WM/0224) on 20th July 2015. Site-specific approvals have been obtained from NHS Research, Development and Innovation departments.

Dissemination policy

The study team are committed to full disclosure of the results of the trial. Findings will be reported in accordance with CONSORT guidelines (30) and we aim to publish in high impact journals. Our patient representatives will assist with dissemination of study results through INVOLVE, other cancer patient groups and organisations including www.independentcancerpatientsvoice.org.uk. The funder will take no role in the analysis or interpretation of trial results.

Discussion

The PROSPER trial will be the largest UK RCT examining the effectiveness of an early, supervised exercise and behavioural support intervention for women at risk of developing shoulder problems after breast cancer surgery. Previous trials in this field have been criticised for being of poor methodological quality and lacking in important outcome measures, such as patient-reported shoulder function and health-related quality of life. PROSPER will provide empirical data on whether a physiotherapy-led exercise programme is effective for reducing shoulder disability when delivered in a pragmatic NHS clinical setting. The design and development of this complex intervention was underpinned by multiple stages of work, in line with MRC guidance on the development of complex interventions. A full description of the PROSPER exercise intervention has been submitted elsewhere for publication.

For peer review only - http://bmjopen.bmj.com/site/about/guidelines.xhtml

Acknowledgements

We extend very grateful thanks to all the trial participants. We are also grateful to all the physiotherapy staff, surgical oncology teams, breast cancer nurses and research departments collaborating on this study.

Collaborators

PROSPER Study Group: Chief Investigator: Professor Julie Bruce. Co-investigators (Grant holders): Professor Sarah E Lamb, Dr Esther Williamson, Dr Ranjit Lall, Professor Stavros Petrou, Mr Alastair M Thompson, Dr John Williams and Dr Catherine Harkin (deceased).

Trial Coordination/Administration: Mrs Emma J Withers, Mrs Lauren Betteley, Mr Craig Turner, Mrs Loraine Chowdhury.

Senior Project Managers: Mrs Susie Hennings, Mrs Helen Higgins.

Research Fellows/Associates: Dr Helen Richmond, Mrs Clare Lait (Physiotherapist), Dr Sophie Rees (Qualitative), Mr Pankaj Mistry (Medical Statistics), Dr Alastair Canaway (Health Economics).

Patient representatives: Dr Catherine Harkin (Deceased), Mrs Marie van Laar, Mrs Lyn Ankcorn.

Surgical Leads: Miss Abigail Tomlins, Miss Raghavan Vidya, Ms Pankaj G Roy, Miss Kat McEvoy, Miss Rachel Soulsby.

Intervention development: Mrs Clare Lait, Dr Esther Williamson, Dr Cynthia Srikesavan, Mrs Jane Moser, Dr Meredith Newman, Dr Sophie Rees, Mrs Lauren Betteley, Dr Helen Richmond, Dr Beth Fordham, Professor Sarah E Lamb and Professor Julie Bruce.

Data Programming team: Mr Ade Willis, Mr Henry Adjei.

Quality Assurance: Ms Claire Daffern.

Contributors: JB obtained study funding with support from SEL, EW, RL, SP and AMT. JB, SEL, EW, CL, RL, AMT, LB, SR and SP participated in the design of the study. EJW and LB coordinate study administration, acquisition of trial data and administrative support (CT/LC). PM will undertake statistical analysis, under direction of RL, senior trial statistician. AC is

For peer review only - http://bmjopen.bmj.com/site/about/guidelines.xhtml

For peer review only

responsible for health economic analysis, supported by SP, senior health economist. JB and HR drafted the manuscript. All authors critically revised the manuscript for intellectual content and approved the final manuscript. This trial protocol is published on behalf of the PROSPER Study Group.

Funding

The PROSPER trial is funded by the National Institute of Health Research Technology Assessment Programme (NiHR HTA), project number 13/84/10. The views expressed in this publication are those of the authors and not necessarily those of the NIHR or Department of Health. This project benefited from facilities funded by Birmingham Science City Translational Medicine Clinical Research and Infrastructure Trials Platform, with support from Advantage West Midlands (AWM). The trial sponsor is the University of Warwick and University Hospitals Coventry and Warwickshire NHS Trust.

Trial Registration:

International Standard Randomised Controlled Trial Number: ISRCTN 35358984.

Competing interests

One author provides private physiotherapy to cancer patients (CL).

Data sharing statement

The trial statisticians and iDMC will have access to the dataset for the analysis of trial outcomes. The CI will have access to the data and take full responsibility for the analysis and publication of results. Once the main analyses have been undertaken, data will be available to other investigators subject to approval of data analysis plans by the steering committee and compliance with the University of Warwick Standard Operating Procedures on Data Management and Sharing. We will comply with Data Sharing Policies that may be instituted by the Funder (NIHR) during the lifetime of the project. The full PROSPER

For peer review only - http://bmjopen.bmj.com/site/about/guidelines.xhtml

intervention manual and related materials will be available for wider access on completion of the main trial, according to funder and institutional repository requirements.

Data Monitoring Committee: Professor Malcolm Reed (Chair), Dr Rhian Gabe, Dr Matthew Maddocks.

Trial Steering Committee: Professor Steven Duffy (Chair), Dr Anna Kirby, Dr Karen Robb.

We dedicate this article to Professor Adele Frances (Deceased) who served on the PROSPER TSC from 2015-2016.

For peer review only

For peer review only - http://bmjopen.bmj.com/site/about/guidelines.xhtml

References

1. CRUK. *Cancer Research UK: Breast Cancer Survival Statistics* [cited 2014. Available from: http://www.cancerresearchuk.org/cancer-info/cancerstats/types/breast/survival/.

2. Mejdahl MK, Andersen KG, Gärtner R, Kroman N, Kehlet H. Persistent pain and sensory disturbances after treatment for breast cancer: six year nationwide follow-up study. BMJ. 2013;346:f1865.

3. McNeely ML, Binkley JM, Pusic AL, Campbell KL, Gabram S, Soballe PW. A prospective model of care for breast cancer rehabilitation: postoperative and postreconstructive issues. Cancer. 2012;118(8 Suppl):2226-36.

4. Lee TS, Kilbreath SL, Refshauge KM, Herbert RD, Beith JM. Prognosis of the upper limb following surgery and radiation for breast cancer. Breast Cancer Res Treat. 2008;110(1):19-37.

5. Bruce J, Thornton AJ, Powell R, Johnston M, Wells M, Heys SD, et al. Psychological, surgical, and sociodemographic predictors of pain outcomes after breast cancer surgery: a population-based cohort study. Pain. 2014;155(2):232-43.

6. Kootstra JJ, Dijkstra PU, Rietman H, de Vries J, Baas P, Geertzen JH, et al. A longitudinal study of shoulder and arm morbidity in breast cancer survivors 7 years after sentinel lymph node biopsy or axillary lymph node dissection. Breast Cancer Res Treat. 2013;139(1):125-34.

7. Meretoja TJ, Andersen KG, Bruce J, Haasio L, Sipila R, Scott NW, et al. Clinical Prediction Model and Tool for Assessing Risk of Persistent Pain After Breast Cancer Surgery. J Clin Oncol. 2017;35(15):1660-7.

8. DiSipio T, Rye S, Newman B, Hayes S. Incidence of unilateral arm lymphoedema after breast cancer: a systematic review and meta-analysis. The Lancet Oncology. 2013;14(6):500-15.

For peer review only - http://bmjopen.bmj.com/site/about/guidelines.xhtml

9. McNeely ML, Campbell K, Ospina M, Rowe BH, Dabbs K, Klassen TP, et al. Exercise interventions for upper-limb dysfunction due to breast cancer treatment. The Cochrane database of systematic reviews. 2010(6):CD005211.

10. Todd J, Scally A, Dodwell D, Horgan K, Topping A. A randomised controlled trial of 0two programmes of shoulder exercise following axillary node dissection for invasive breast cancer. Physiotherapy. 2008;94(4):265-73.

11. Chan A-W, Tetzlaff JM, Gøtzsche PC, Altman DG, Mann H, Berlin JA, et al. SPIRIT 2013 explanation and elaboration: guidance for protocols of clinical trials. Bmj. 2013;346:e7586.

12. Department of Health (DH). Toolkit for producing patient information. London, The Department of Health; 2003.

13. BCC. Breast Cancer Campaign [Available from: http://www.breastcancercare.org.uk/.

14. Harrington S, Michener LA, Kendig T, Miale S, George SZ. Patient-reported upper extremity outcome measures used in breast cancer survivors: a systematic review. Archives of physical medicine and rehabilitation. 2014;95(1):153-62.

15. Dixon D, Johnston M, McQueen M, Court-Brown C. The Disabilities of the Arm, Shoulder and Hand Questionnaire (DASH) can measure the impairment, activity limitations and participation restriction constructs from the International Classification of Functioning, Disability and Health (ICF). BMC musculoskeletal disorders. 2008;9:114.

16. Beurskens CH, van Uden CJ, Strobbe LJ, Oostendorp RA, Wobbes T. The efficacy of physiotherapy upon shoulder function following axillary dissection in breast cancer, a randomized controlled study. BMC cancer. 2007;7:166.

17. Sorensen AA, Howard D, Tan WH, Ketchersid J, Calfee RP. Minimal clinically important differences of 3 patient-rated outcomes instruments. The Journal of hand surgery. 2013;38(4):641-9.

For peer review only
For peer review only - http://bmjopen.bmj.com/site/about/guidelines.xhtml

18. Dunn G, Maracy M, Tomenson B. Estimating treatment effects from randomized clinical trials with noncompliance and loss to follow-up: the role of instrumental variable methods. Statistical methods in medical research. 2005;14(4):369-95.

19. Perneger TV, Burnand B. A simple imputation algorithm reduced missing data in SF-12 health surveys. Journal of clinical epidemiology. 2005;58(2):142-9.

20. Knox CR, Lall R, Hansen Z, Lamb SE. Treatment compliance and effectiveness of a cognitive behavioural intervention for low back pain: a complier average causal effect approach to the BeST data set. BMC musculoskeletal disorders. 2014;15:17.

21. Williams MA, Williamson EM, Heine PJ, Nichols V, Glover MJ, Dritsaki M, et al. Strengthening And stretching for Rheumatoid Arthritis of the Hand (SARAH). A randomised controlled trial and economic evaluation. Health Technol Assess. 2015;19(19):1-222.

22. NICE. *Guide to the Methods of Technology Appraisal* 2013 [Available from: http://publications.nice.org.uk/guide-to-the-methods-of-technology-appraisal-2013-pmg9.

23. Hollis S, Campbell F. What is meant by intention to treat analysis? Survey of published randomised controlled trials. Bmj. 1999;319(7211):670-4.

24. Dolan P. Modeling valuations for EuroQol health states. Medical care. 1997;35(11):1095-108.

25. van Hout B, Janssen MF, Feng YS, Kohlmann T, Busschbach J, Golicki D, et al. Interim scoring for the EQ-5D-5L: mapping the EQ-5D-5L to EQ-5D-3L value sets. Value in health : the journal of the International Society for Pharmacoeconomics and Outcomes Research. 2012;15(5):708-15.

26. Herdman M, Gudex C, Lloyd A, Janssen M, Kind P, Parkin D, et al. Development and preliminary testing of the new five-level version of EQ-5D (EQ-5D-5L). Quality of life research : an international journal of quality of life aspects of treatment, care and rehabilitation. 2011;20(10):1727-36.

27. Brazier J, Roberts J, Deverill M. The estimation of a preference-based measure of health from the SF-36. Journal of health economics. 2002;21(2):271-92.

For peer review only

For peer review only - http://bmjopen.bmj.com/site/about/guidelines.xhtml

28. Devlin N, Shah K, Feng Y, Mulhern B, van Hout B. Valuing health-related quality of life: an EQ-5D-5L value set for England. 2016.

29. Sculpher MJ, Claxton K, Drummond M, McCabe C. Whither trial-based economic evaluation for health care decision making? Health economics. 2006;15(7):677-87.

30. Moher D, Schulz KF, Altman DG. The CONSORT statement: revised recommendations for improving the quality of reports of parallel-group randomised trials. Lancet. 2001;357(9263):1191-4.

For peer review only

Figure 1 Study outcome measures and assessment time points

Figure 2 Trial flow diagram

For peer review only

For peer review only - http://bmjopen.bmj.com/site/about/guidelines.xhtml

Table 1 World Health Organisation Trial Registration Data Set

**Data category**	**Information**
Primary registry and trial identifying number	ISRCTN35358984
Date of registration in primary registry	Project Number 13/84/10
Secondary identifying numbers	HTA
Source of monetary or material support	National Institute for Health Research (NIHR) Health Technology Assessment (HTA)
Joint sponsor	University of Warwick / University Hospitals Coventry & Warwickshire NHS Trust
Contact for public queries	prosper@warwick.ac.uk
Contact for scientific queries	Prof Julie Bruce, Warwick Clinical Trials Unit, University of Warwick
Public Title	Exercise to prevent shoulder problems in patients undergoing breast cancer treatment
Scientific Title	The PRevention Of Shoulder ProblEms TRial (PROSPER): a randomised controlled clinical trial comparing physiotherapy-led exercise versus usual care in women at high risk of shoulder problems after breast cancer surgery
Countries of recruitment	UK
Health condition or problem studied	Breast cancer
Interventions	Advice only: Breast Cancer Care leaflets<br>Comparator: Physiotherapy-led structured exercise programme incorporating behavioural

For peer review only - http://bmjopen.bmj.com/site/about/guidelines.xhtml

	strategies
Key inclusion and exclusion criteria	Age: 18 years or over, no upper age restriction<br>Sex: Female<br>Inclusion: confirmed invasive/non-invasive primary breast cancer schedule for surgical excision, at high risk of shoulder problems as defined by criteria given in Table 2.<br>Exclusion: males, and women with exclusion criteria as described in Table 2.
Study type	Interventional<br>Allocation: randomised; individual assignment.<br>Primary purpose: prevention.<br>Phase III
Date of first enrolment	January 2016
Target sample size	350
Recruitment status	Recruiting to July 2017
Primary outcome	Arm, shoulder and hand function as measured using the Disabilities of the Arm, Shoulder and Hand (DASH) questionnaire at 12 months
Key secondary outcomes	DASH subscales, pain (acute, chronic, neuropathic), health-related quality of life, surgical site infection, lymphoedema and other complications, health care resource use.

For peer review only - http://bmjopen.bmj.com/site/about/guidelines.xhtml

	Exercise/activity data to inform adherence to interventions.

For peer review only

For peer review only - http://bmjopen.bmj.com/site/about/guidelines.xhtml

Table 2. Trial inclusion and exclusion criteria

**Inclusion Criteria**	**Exclusion Criteria**
Aged ≥18 years old	Males
Histologically confirmed invasive or non-invasive primary breast cancer scheduled for surgical excision of breast cancer	Women having immediate reconstructive surgery
Considered high risk of developing shoulder problems after surgery defined by one or more of the following:<br>• Planned axillary node clearance<br>• Planned radiotherapy to the axilla and/or supraclavicular*<br>• Existing shoulder problems (based upon PROSPER screening criteria)	Women having sentinel lymph node biopsy (SLNB), with or without breast surgery, unless they fulfil other high risk criteria
Obesity defined as BMI >30	Women having bilateral breast surgery
Any subsequent axillary surgery related to primary surgery e.g. ANC conducted after sentinel lymph node biopsy (SLNB)	Evidence of metastatic disease at time of recruitment
Able to provide written informed consent	
Willing and able to comply with the protocol	
**includes women informed of need for radiotherapy to the axilla and/or supraclavicular within six weeks of surgery, thus potential late entry to the trial is allowed in this setting*	

For peer review only

Table 3. Outcome assessment

Outcome	Domain	Scale / measure	T_0 baseline	t_1 6 weeks	t_2 6 months	t_3 12 months
Primary	Function	DASH				√
Secondary	Function	DASH subscales	√		√	√
	Acute & chronic pain<br>Neuropathic pain	FACT-B+4; NRS<br>DN4	√<br>√	√<br>√	√<br>√	√<br>√
	Complications	SSI + self-report		√	√	√
	Lymphoedema	Self-report	√	√	√	√
	Health-related QoL	SF12 / EQ-5D-5L	√		√	√
	Resource use	Self-report			√	√
	General activity & exercise	PASE items	√	√	√	√

DASH: Disabilities of Arm, Shoulder and Hand questionnaire; DN4: Doleur Neuropathique; SF12: Short Form-12; SSI: surgical site infection; EQ-5D-5L: Euroqol; t: time point; QoL: Quality of Life; PASE: Physical Activity Scale for the Elderly.

30

For peer review only

Figure 1. Study outcome measures and assessment time points

	Study period				
	**Enrolment**	**Allocation**	**Post-allocation**		
**Time point**	*-t₁*	*0*	t_1 6 weeks	t_2 6 months	t_3 12 months
Enrolment:					
Eligibility screen	√				
Informed consent	√				
Randomisation	350	√			
Interventions					
Usual Care (UC)	175	All participants	←→		
UC + PROSPER intervention	175		←	—	→

Figure 1 Study outcome measures and assessment time points

87x46mm (300 x 300 DPI)

review only

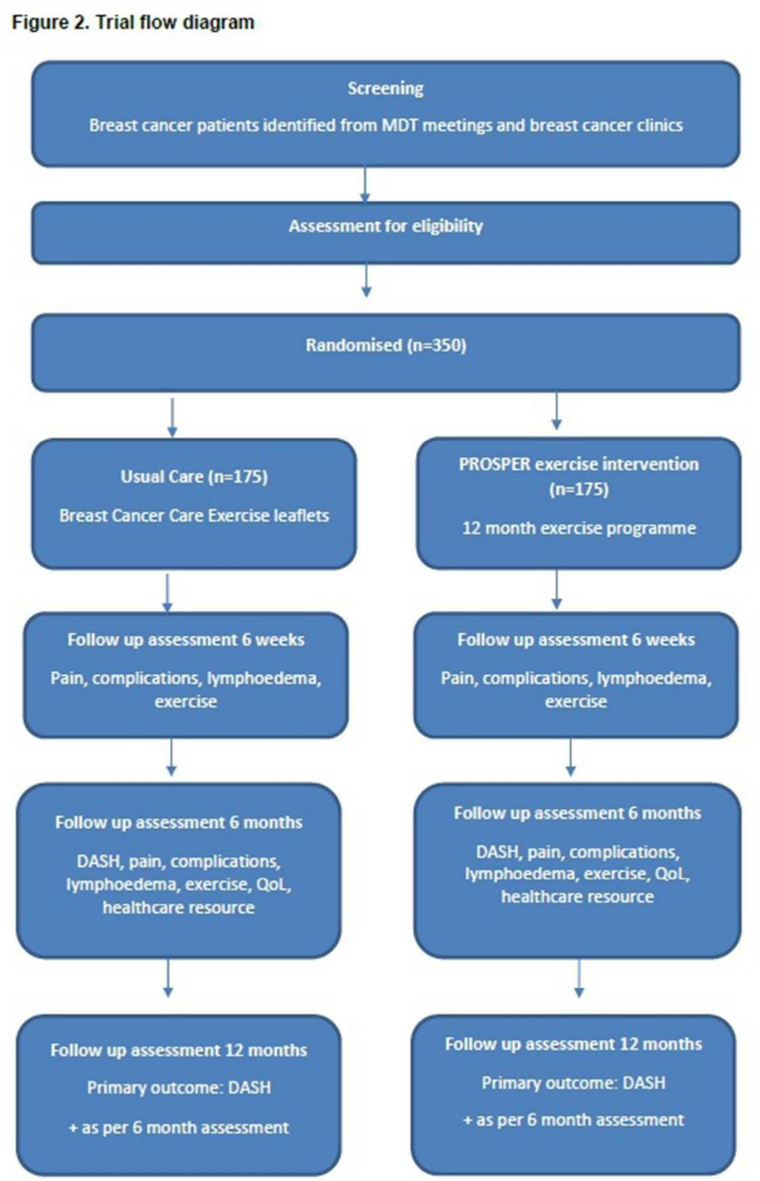

Figure 2 Trial flow diagram

50x65mm (300 x 300 DPI)

SPIRIT

STANDARD PROTOCOL ITEMS: RECOMMENDATIONS FOR INTERVENTIONAL TRIALS

SPIRIT 2013 Checklist: Recommended items to address in a clinical trial protocol and related documents*

Section/item	Item No	Description	Addressed on page number
**Administrative information**			
Title	1	Descriptive title identifying the study design, population, interventions, and, if applicable, trial acronym	1
Trial registration	2a	Trial identifier and registry name. If not yet registered, name of intended registry	4
	2b	All items from the World Health Organization Trial Registration Data Set	Table 1
Protocol version	3	Date and version identifier	3 & 22
Funding	4	Sources and types of financial, material, and other support	25 & Table 1
Roles and responsibilities	5a	Names, affiliations, and roles of protocol contributors	24-25
	5b	Name and contact information for the trial sponsor	Table 1
	5c	Role of study sponsor and funders, if any, in study design; collection, management, analysis, and interpretation of data; writing of the report; and the decision to submit the report for publication, including whether they will have ultimate authority over any of these activities	25-6
	5d	Composition, roles, and responsibilities of the coordinating centre, steering committee, endpoint adjudication committee, data management team, and other individuals or groups overseeing the trial, if applicable (see Item 21a for data monitoring committee)	24

For peer review only - http://bmjopen.bmj.com/site/about/guidelines.xhtml

**Introduction**			
Background and rationale	6a	Description of research question and justification for undertaking the trial, including summary of relevant studies (published and unpublished) examining benefits and harms for each intervention	5 - 7
	6b	Explanation for choice of comparators	10 – 12
Objectives	7	Specific objectives or hypotheses	8
Trial design	8	Description of trial design including type of trial (eg, parallel group, crossover, factorial, single group), allocation ratio, and framework (eg, superiority, equivalence, noninferiority, exploratory)	8 - 9
**Methods: Participants, interventions, and outcomes**			
Study setting	9	Description of study settings (eg, community clinic, academic hospital) and list of countries where data will be collected. Reference to where list of study sites can be obtained	8
Eligibility criteria	10	Inclusion and exclusion criteria for participants. If applicable, eligibility criteria for study centres and individuals who will perform the interventions (eg, surgeons, psychotherapists)	8 – 9
Interventions	11a	Interventions for each group with sufficient detail to allow replication, including how and when they will be administered	10-12 + separate intervention papers
	11b	Criteria for discontinuing or modifying allocated interventions for a given trial participant (eg, drug dose change in response to harms, participant request, or improving/worsening disease)	22
	11c	Strategies to improve adherence to intervention protocols, and any procedures for monitoring adherence (eg, drug tablet return, laboratory tests)	12
	11d	Relevant concomitant care and interventions that are permitted or prohibited during the trial	12
Outcomes	12	Primary, secondary, and other outcomes, including the specific measurement variable (eg, systolic blood pressure), analysis metric (eg, change from baseline, final value, time to event), method of aggregation (eg, median, proportion), and time point for each outcome. Explanation of the clinical relevance of chosen efficacy and harm outcomes is strongly recommended	Table 2
Participant timeline	13	Time schedule of enrolment, interventions (including any run-ins and washouts), assessments, and visits for participants. A schematic diagram is highly recommended (see Figure)	Figure 1

For peer review only - http://bmjopen.bmj.com/site/about/guidelines.xhtml

Sample size	14	Estimated number of participants needed to achieve study objectives and how it was determined, including clinical and statistical assumptions supporting any sample size calculations	16
Recruitment	15	Strategies for achieving adequate participant enrolment to reach target sample size	9
**Methods: Assignment of interventions (for controlled trials)**			
Allocation:			
Sequence generation	16a	Method of generating the allocation sequence (eg, computer-generated random numbers), and list of any factors for stratification. To reduce predictability of a random sequence, details of any planned restriction (eg, blocking) should be provided in a separate document that is unavailable to those who enrol participants or assign interventions	9
Allocation concealment mechanism	16b	Mechanism of implementing the allocation sequence (eg, central telephone; sequentially numbered, opaque, sealed envelopes), describing any steps to conceal the sequence until interventions are assigned	9
Implementation	16c	Who will generate the allocation sequence, who will enrol participants, and who will assign participants to interventions	10
Blinding (masking)	17a	Who will be blinded after assignment to interventions (eg, trial participants, care providers, outcome assessors, data analysts), and how	10
	17b	If blinded, circumstances under which unblinding is permissible, and procedure for revealing a participant's allocated intervention during the trial	10
**Methods: Data collection, management, and analysis**			
Data collection methods	18a	Plans for assessment and collection of outcome, baseline, and other trial data, including any related processes to promote data quality (eg, duplicate measurements, training of assessors) and a description of study instruments (eg, questionnaires, laboratory tests) along with their reliability and validity, if known. Reference to where data collection forms can be found, if not in the protocol	11 & Table 2
	18b	Plans to promote participant retention and complete follow-up, including list of any outcome data to be collected for participants who discontinue or deviate from intervention protocols	11 & 14

For peer review only - http://bmjopen.bmj.com/site/about/guidelines.xhtml

Data management	19	Plans for data entry, coding, security, and storage, including any related processes to promote data quality (eg, double data entry; range checks for data values). Reference to where details of data management procedures can be found, if not in the protocol	20
Statistical methods	20a	Statistical methods for analysing primary and secondary outcomes. Reference to where other details of the statistical analysis plan can be found, if not in the protocol	17-20
	20b	Methods for any additional analyses (eg, subgroup and adjusted analyses)	17-20
	20c	Definition of analysis population relating to protocol non-adherence (eg, as randomised analysis), and any statistical methods to handle missing data (eg, multiple imputation)	18-20
**Methods: Monitoring**			
Data monitoring	21a	Composition of data monitoring committee (DMC); summary of its role and reporting structure; statement of whether it is independent from the sponsor and competing interests; and reference to where further details about its charter can be found, if not in the protocol. Alternatively, an explanation of why a DMC is not needed	22
	21b	Description of any interim analyses and stopping guidelines, including who will have access to these interim results and make the final decision to terminate the trial	20
Harms	22	Plans for collecting, assessing, reporting, and managing solicited and spontaneously reported adverse events and other unintended effects of trial interventions or trial conduct	22
Auditing	23	Frequency and procedures for auditing trial conduct, if any, and whether the process will be independent from investigators and the sponsor	12 & described in intervention papers
**Ethics and dissemination**			
Research ethics approval	24	Plans for seeking research ethics committee/institutional review board (REC/IRB) approval	22
Protocol amendments	25	Plans for communicating important protocol modifications (eg, changes to eligibility criteria, outcomes, analyses) to relevant parties (eg, investigators, REC/IRBs, trial participants, trial registries, journals, regulators)	4

For peer review only - http://bmjopen.bmj.com/site/about/guidelines.xhtml

Consent or assent	26a	Who will obtain informed consent or assent from potential trial participants or authorised surrogates, and how (see Item 32)	9
	26b	Additional consent provisions for collection and use of participant data and biological specimens in ancillary studies, if applicable	9 & 21
Confidentiality	27	How personal information about potential and enrolled participants will be collected, shared, and maintained in order to protect confidentiality before, during, and after the trial	9
Declaration of interests	28	Financial and other competing interests for principal investigators for the overall trial and each study site	24
Access to data	29	Statement of who will have access to the final trial dataset, and disclosure of contractual agreements that limit such access for investigators	26
Ancillary and post-trial care	30	Provisions, if any, for ancillary and post-trial care, and for compensation to those who suffer harm from trial participation	12
Dissemination policy	31a	Plans for investigators and sponsor to communicate trial results to participants, healthcare professionals, the public, and other relevant groups (eg, via publication, reporting in results databases, or other data sharing arrangements), including any publication restrictions	3-4 & 20
	31b	Authorship eligibility guidelines and any intended use of professional writers	3
	31c	Plans, if any, for granting public access to the full protocol, participant-level dataset, and statistical code	24
**Appendices**			
Informed consent materials	32	Model consent form and other related documentation given to participants and authorised surrogates	Can be provided as Appendix if requested
Biological specimens	33	Plans for collection, laboratory evaluation, and storage of biological specimens for genetic or molecular analysis in the current trial and for future use in ancillary studies, if applicable	Not applicable

*It is strongly recommended that this checklist be read in conjunction with the SPIRIT 2013 Explanation & Elaboration for important clarification on the items. Amendments to the protocol should be tracked and dated. The SPIRIT checklist is copyrighted by the SPIRIT Group under the Creative Commons "Attribution-NonCommercial-NoDerivs 3.0 Unported" license.

For peer review only - http://bmjopen.bmj.com/site/about/guidelines.xhtml

BMJ Open

A randomised controlled trial of exercise to prevent shoulder problems in women undergoing breast cancer treatment: study protocol for the Prevention of Shoulder Problems Trial (UK PROSPER Trial)

Journal:	*BMJ Open*
Manuscript ID	bmjopen-2017-019078.R2
Article Type:	Protocol
Date Submitted by the Author:	04-Jan-2018
Complete List of Authors:	Bruce, Julie; University of Warwick, Warwick Clinical Trials Unit<br>Williamson, Esther; Nuffield Department of Orthopaedics, Rheumatology and Musculoskeletal Sciences, University of Oxford, United Kingdom. , Centre for Rehabilitation Research in Oxford<br>Lait, Clare; Gloucestershire Care Services NHS Trust<br>Richmond, Helen ; University of Warwick, Warwick Clinical Trials Unit<br>Betteley, Lauren; University of Warwick Warwick Medical School, Warwick Clinical Trials Unit<br>Lall, Ranjit; University of Warwick, Warwick Clinical Trials Unit<br>Petrou, Stavros; University of Warwick, Warwick Medical School<br>Rees, Sophie; University of Warwick, Warwick Clinical Trials Unit<br>Withers, Emma; University of Warwick, Warwick Clinical Trials Unit<br>Lamb, Sarah; University of Oxford, Nuffield Department of Orthopaedics, Rheumatology and Musculoskeletal Sciences<br>Thompson, Alastair; University of Texas M.D. Anderson Cancer Center, Department of Breast Surgical Oncology and Department of Translational Molecular Pathology
**Primary Subject Heading**:	Rehabilitation medicine
Secondary Subject Heading:	Surgery, Health services research, Oncology
Keywords:	Breast surgery < SURGERY, Clinical trials < THERAPEUTICS, Rehabilitation medicine < INTERNAL MEDICINE

SCHOLARONE™
Manuscripts

A randomised controlled trial of exercise to prevent shoulder problems in women undergoing breast cancer treatment: study protocol for the Prevention of Shoulder Problems Trial (UK PROSPER Trial)

Authors: Julie Bruce[1], Esther Williamson[2], Clare Lait[3], Helen Richmond[1], Lauren Betteley[1], Ranjit Lall[1], Stavros Petrou[1], Sophie Rees[1], Emma J Withers[1], Sarah E Lamb[1,2] and Alastair Thompson[4] on behalf of the PROSPER Study Group.

Author affiliations

1. Warwick Clinical Trials Unit, Division of Health Sciences, University of Warwick, Coventry CV4 7AL, UK
2. Nuffield Department of Orthopaedics Rheumatology & Musculoskeletal Sciences, University of Oxford, Windmill Road, Oxford, OX3 7LD, UK
3. Gloucestershire Care Services NHS Trust, 1010 Gloucester Business Park, Pioneer Avenue, Brockworth, Gloucester, GL3 4AW, UK
4. Department of Breast Surgical Oncology & Department of Translational Molecular Pathology, University of Texas M.D. Anderson Cancer Center, 1400 Pressler Drive, Houston, Texas, US

Corresponding author: *Professor Julie Bruce; julie.bruce@warwick.ac.uk*

For peer review only

Abstract

Musculoskeletal shoulder problems are common after breast cancer treatment. Early postoperative exercises targeting the upper limb may improve shoulder function. This protocol describes a National Institute for Health Research (NIHR) funded randomised controlled trial (RCT) to evaluate the clinical and cost-effectiveness of an early supervised structured exercise programme compared to usual care, for women at high risk of developing shoulder problems after breast cancer surgery.

Methods

This pragmatic two-armed, multicentre RCT is underway within secondary care in the United Kingdom (UK). PROSPER aims to recruit 350 women from approximately 15 UK centres with follow-up at 6 weeks, 6 and 12 months after randomisation. Recruitment processes and intervention development were optimised through qualitative research during a six-month internal pilot phase. Participants are randomised to the PROSPER intervention or best practice usual care only. The PROSPER intervention is delivered by physiotherapists and incorporates three main components: shoulder-specific exercises targeting range of movement and strength; general physical activity; and behavioural strategies to encourage adherence and support exercise behaviour. The primary outcome is upper arm function assessed using the Disabilities of the Arm Shoulder and Hand (DASH) questionnaire at 12 months post-randomisation. Secondary outcomes include DASH subscales, acute and chronic pain, complications, health related quality of life, and healthcare resource use. We will interview a subsample of twenty participants to explore their experiences of the trial interventions.

Discussion

The PROSPER study is the first multicentre UK clinical trial to investigate the clinical and cost-effectiveness of supported exercise in the prevention of shoulder problems in high risk women undergoing breast cancer surgery. The findings will inform future clinical practice and provide valuable insight into the role of physiotherapy-supported exercise in breast cancer rehabilitation.

For peer review only - http://bmjopen.bmj.com/site/about/guidelines.xhtml

Trial registration: ISRCTN35358984

Protocol version: Version 2.1; dated 11/01/2017

Funding: NIHR HTA (Project Number 13/84/10)

Strengths and limitations of this study

Strengths:

- A large pragmatic study delivering a complex intervention to prevent postoperative health problems in newly diagnosed cancer patients within secondary care;
- A strength of the evaluation is the mixed methods approach incorporating embedded qualitative research and economic analysis

Limitations:

- Recruited participants undergo multiple cancer treatments thus experience a complicated postoperative recovery pathway.

For peer review only

For peer review only - http://bmjopen.bmj.com/site/about/guidelines.xhtml

Background

Breast cancer is the most common cancer in women in the UK with a 30% increase in incidence since the early 1970s (1). Due to advances in screening and treatment, the survival rate has progressively risen and now two thirds of women will survive for 20 years beyond their breast cancer diagnosis (1). The mainstay treatment is surgery to the breast and axilla, supplemented with chemotherapy, radiotherapy and endocrine therapy, depending upon tumour stage and other clinical criteria. As a consequence of these treatments, upper limb problems such as decreased shoulder range of movement (ROM), impaired strength, chronic pain, sensory disturbances and lymphoedema are common adverse treatment effects (2, 3). Studies suggest that up to 67% of women have arm or shoulder symptoms up to 3 years after treatment (4). Persistent upper limb dysfunction and pain are debilitating, have a negative impact upon sleep, quality of life, physical functioning and emotional wellbeing. Given successes in increasing survival, it is timely to identify strategies to improve the health-related quality of life of women after breast cancer treatment.

Several risk factors for shoulder and upper body problems after breast cancer treatment have been identified, including treatment-related factors, such as type of axillary surgery and radiotherapy (RT), and patient factors such as age, body mass index (BMI), and pre-existing shoulder problems (4, 5). Women undergoing mastectomy compared to breast conserving surgery are at greater risk of postoperative shoulder restrictions (odds ratio (OR) 5.7, 95% confidence interval (CI) 1.03, 31.2) (4). Additionally, those undergoing axillary lymph node clearance (ANC) are at greater risk of postoperative arm complaints compared to those having sentinel lymph node biopsy (SLNB; OR 9.8; 95% CI 3.5, 27.5) (4-6). Radiotherapy (RT), particularly to the axilla or chest wall, increases the odds of shoulder restriction (pooled OR 1.7, 95% CI 1.0, 2.9) and lymphoedema (pooled OR 1.5, 95% CI 1.2, 1.8) compared to women treated without adjuvant RT (4). Women reporting problems in the upper body and shoulder region before surgery are also at increased risk of chronic postoperative pain (5, 7).

For peer review only - http://bmjopen.bmj.com/site/about/guidelines.xhtml

BMI at time of surgery has been shown to have an independent negative effect on shoulder external rotation up to seven years after breast cancer treatment, and increased BMI is a risk factor for chronic postoperative pain and arm lymphoedema (7, 8). It is important that the UK National Health Service (NHS) provides optimal care for these women at high risk of developing shoulder problems to ensure recovery and return to usual activities after cancer treatment.

A Cochrane review identified 24 studies (2132 participants) investigating exercise following breast cancer surgery (9). Six studies (n=354), conducted outside of the UK, found that structured postoperative exercise significantly improved shoulder ROM in the short and long term when compared to usual care (9). Ten studies (n=1304) have evaluated timing of exercise delivery; programmes initiated immediately postoperatively (1-3 days) versus delayed exercise suggest that early postoperative exercise does significantly improve long-term shoulder ROM. However, some studies reported an increased risk of wound-related complications with early exercise, such as seroma and surgical site infection (9). The largest UK trial to date (n=116 patients), published after the Cochrane review, found that participants were less likely to develop lymphoedema when exercises were limited to 90° of shoulder elevation during the first postoperative week compared to those performing unrestricted exercises (10). These previous trials investigating the efficacy of exercise following breast cancer surgery have been criticised for being of poor methodological quality and for omitting important patient-reported outcomes such as function and health-related quality of life (9). Furthermore, there is ongoing uncertainty around the optimal type, dose, and timing of exercise after breast cancer treatment. Moreover, none of the trials conducted to date have investigated the cost-effectiveness of structured exercise programmes after breast cancer treatment.

For peer review only - http://bmjopen.bmj.com/site/about/guidelines.xhtml

Rationale for a trial

To date, no large scale, high-quality, multicentre RCT investigating the clinical effectiveness of a structured physiotherapy intervention compared to usual care for women undergoing breast cancer surgery has been conducted. Given the lack of knowledge regarding the intensity and duration of exercise interventions after breast cancer surgery, this trial will provide evidence on whether a rigorously designed physiotherapy-led intervention, incorporating behaviour change theory, improves postoperative function and related outcomes, and whether this is cost-effective to deliver in the NHS setting.

Methods

Aim

The overall aim of PROSPER is to investigate the clinical and cost-effectiveness of an early supervised exercise programme compared to best practice usual care for women at high risk of shoulder problems after treatment for breast cancer, on outcomes of upper arm function, complications and quality of life. Specific trial objectives are:

1. To develop and refine a complex intervention of physiotherapy-led exercise for women at risk of developing musculoskeletal problems after breast cancer treatment;
2. To assess the acceptability of the structured exercise programme and outcome measures, optimise participant recruitment and refine trial processes during a six-month internal pilot phase at three clinical centres;
3. Use findings from the internal pilot phase to undertake a definitive full RCT in approximately 15 UK NHS breast cancer centres.

This protocol follows guidance from the Standard Protocol Items: Recommendations for Interventional Trials (SPIRIT)(11). Core trial information is presented in Table 1 (WHO Trial Registration Data Set). Figure 1 details the schedule of enrolment, interventions and assessment.

For peer review only - http://bmjopen.bmj.com/site/about/guidelines.xhtml

Table 1 World Health Organisation Trial Registration Data Set

**Data category**	**Information**
Primary registry and trial identifying number	ISRCTN35358984
Date of registration in primary registry	Project Number 13/84/10
Secondary identifying numbers	HTA
Source of monetary or material support	National Institute for Health Research (NIHR) Health Technology Assessment (HTA)
Joint sponsor	University of Warwick / University Hospitals Coventry & Warwickshire NHS Trust
Contact for public queries	prosper@warwick.ac.uk
Contact for scientific queries	Prof Julie Bruce, Warwick Clinical Trials Unit, University of Warwick
Public Title	Exercise to prevent shoulder problems in patients undergoing breast cancer treatment
Scientific Title	The PRevention Of Shoulder ProblEms TRial (PROSPER): a randomised controlled clinical trial comparing physiotherapy-led exercise versus usual care in women at high risk of shoulder problems after breast cancer surgery
Countries of recruitment	UK
Health condition or problem studied	Breast cancer
Interventions	Advice only: Breast Cancer Care leaflets<br>Comparator: Physiotherapy-led structured exercise programme incorporating behavioural

For peer review only - http://bmjopen.bmj.com/site/about/guidelines.xhtml

	strategies
Key inclusion and exclusion criteria	Age: 18 years or over, no upper age restriction<br>Sex: Female<br>Inclusion: confirmed invasive/non-invasive primary breast cancer schedule for surgical excision, at high risk of shoulder problems as defined by criteria given in Table 2.<br>Exclusion: males, and women with exclusion criteria as described in Table 2.
Study type	Interventional<br>Allocation: randomised; individual assignment.<br>Primary purpose: prevention.<br>Phase III
Date of first enrolment	January 2016
Target sample size	350
Recruitment status	Recruiting to July 2017
Primary outcome	Arm, shoulder and hand function as measured using the Disabilities of the Arm, Shoulder and Hand (DASH) questionnaire at 12 months
Key secondary outcomes	DASH subscales, pain (acute, chronic, neuropathic), health-related quality of life, surgical site infection, lymphoedema and other complications, health care resource use.

For peer review only - http://bmjopen.bmj.com/site/about/guidelines.xhtml

	Exercise/activity data to inform adherence to interventions.

For peer review only

For peer review only - http://bmjopen.bmj.com/site/about/guidelines.xhtml

Trial design and setting

A multicentre, pragmatic, parallel, two-arm RCT with an internal pilot study and embedded economic evaluation and qualitative studies. The trial framework is superiority rather than equivalence or exploratory. The trial is currently open and recruiting from 17 NHS tertiary breast cancer centres across England. Participants are randomised in a 1:1 ratio between intervention and control arms.

Patient and public involvement (PPI) in trial design

Four female PPI representatives, all of whom were treated for breast cancer, were consulted during the initial grant preparation, intervention development and trial set up. Our PPI representatives contributed to the design of the intervention and advised on recruitment-related issues; they provided valuable insight into the worries and concerns experienced during cancer treatment.

Eligibility Criteria

Women are eligible to participate in PROSPER if they are: diagnosed with histologically confirmed invasive or non-invasive primary breast cancer scheduled for surgical excision; aged 18 years or over; can comply with the protocol; willing to provide written informed consent; and considered as being at high risk of developing postoperative shoulder problems (Table 2). This is a pragmatic trial and it is important that inclusion criteria reflect contemporary clinical practice. Therefore, women are also eligible where a later decision is made for postoperative radiotherapy (RT) to the axilla and/or supraclavicular region, thus changing their risk status from low to high. ‘Late entry’ women are eligible for the trial if the decision for postoperative RT is made within six weeks of surgery. Women who have had previous breast surgery (such as excision of a benign tumour or breast cyst) and those women who have had previous contralateral (opposite side) mastectomy, are eligible for invitation providing they fulfil high risk criteria for shoulder problems. Women having immediate reconstruction or bilateral breast surgery are ineligible as the usual NHS

For peer review only - http://bmjopen.bmj.com/site/about/guidelines.xhtml

postoperative care pathway often includes routine postoperative physiotherapy. Exclusion criteria are presented in Table 2.

Table 2. Trial inclusion and exclusion criteria

**Inclusion Criteria**	**Exclusion Criteria**
Aged ≥18 years old	Males
Histologically confirmed invasive or non-invasive primary breast cancer scheduled for surgical excision of breast cancer	Women having immediate reconstructive surgery
Considered high risk of developing shoulder problems after surgery defined by one or more of the following:<br>• Planned axillary node clearance<br>• Planned radiotherapy to the axilla and/or supraclavicular*<br>• Existing shoulder problems (based upon PROSPER screening criteria)	Women having sentinel lymph node biopsy (SLNB), with or without breast surgery, unless they fulfil other high risk criteria
Obesity defined as BMI >30	Women having bilateral breast surgery
Any subsequent axillary surgery related to primary surgery e.g. ANC conducted after sentinel lymph node biopsy (SLNB)	Evidence of metastatic disease at time of recruitment
Able to provide written informed consent	
Willing and able to comply with the protocol	
**includes women informed of need for radiotherapy to the axilla and/or supraclavicular within six weeks of surgery, thus potential late entry to the trial is allowed in this setting*	

Participant screening, recruitment and consent

Participants are screened and identified from multi-disciplinary team (MDT) meetings and preoperative breast/oncology clinic lists in secondary care. The initial screening process is undertaken by a member of the clinical team, research nurse or trained designee. Potentially

For peer review only - http://bmjopen.bmj.com/site/about/guidelines.xhtml

eligible patients are approached by clinical or research staff and are given a Patient Information Sheet (PIS) with further explanation of the trial. Figure 2 summarises participant flow.

Allocation sequence generation and randomisation

Randomisation is based upon a computer-generated algorithm held and controlled centrally by the Warwick Clinical Trials Unit (WCTU) programming team, independent from the PROSPER trial team. The WCTU telephone randomisation service is used whereby randomisation occurs after eligibility and informed consent has been obtained. Concealment of allocation is maintained. An automated confirmation email of intervention allocation is generated to the study team. Randomisation is stratified by the following variables: (i) first versus repeat surgery; (ii) centre; and (iii) whether informed of the need for radiotherapy within six weeks of surgery. The first variable adjusts for the requirement for any additional surgery which may change risk status from low to high (e.g. second procedure ANC or reexcision of surgical margins). The second stratification variable ensures balanced allocation across each recruitment site. The third variable accounts for late entry to the trial, thus relates to the timing of intervention delivery and whether participants are randomised preoperatively (up to the day of surgery) or within the first six weeks postoperatively. Due to the nature of the study intervention, it is not possible to blind participants or treating physiotherapists to treatment allocation. However, receipt and handling of outcome data collection is blinded, thus data entry of returned postal questionnaires, data cleaning and interim statistical analyses are conducted without knowledge of treatment allocation (blinded).

Interventions

Control Arm: Usual Care

All participants allocated to the usual care arm receive best practice usual care in the form of written leaflets containing information about exercises, recovery after surgery, and

For peer review only - http://bmjopen.bmj.com/site/about/guidelines.xhtml

treatments for breast cancer. During the pilot phase, different exercise information leaflets were reviewed and considered; we also consulted best practice guidance for written patient information materials (12). The most commonly used information leaflets were ‘Exercises after Breast Cancer Surgery (BCC6)’ and ‘Your Operation and Recovery (BCC151)’ published by Breast Cancer Care (BCC) (13). The BCC leaflets were selected because of content, style and clarity of presentation of information. These two information leaflets were given to all patients before surgery by breast care nurses, or other healthcare professionals, depending upon local practice.

Intervention Arm: PROSPER exercise programme

Participants randomised to the active intervention receive usual care leaflets in addition to the PROSPER intervention: a structured individualised exercise programme, comprising a minimum of three face-to-face and maximum of six sessions or contacts with a physiotherapist. As per Medical Research Council and TiDieR guidance, a more detailed description of the intervention development and final content has been described separately (submitted for publication). . We selected exercises and components based upon systematic reviews and clinical guidelines. A Cochrane review investigated the effectiveness of exercise interventions in preventing, minimising or improving upper-limb dysfunction due to breast cancer treatment (9) . This review included 24 trials and classified exercise type as active, active-assisted, passive range of movement, manual stretching, active stretching and resistance exercises. We considered these components in relation to evidence of effectiveness on shoulder range of movement and strength. This process was also augmented by eliciting opinions from clinical experts in the field of cancer rehabilitation and health psychology. The final PROSPER programme comprises specific exercises targeting shoulder range of motion and upper arm muscle strength, general physical activity, and behavioural adherence strategies.

For peer review only - http://bmjopen.bmj.com/site/about/guidelines.xhtml

Overview of exercise intervention

The intervention is predominantly delivered in physiotherapy outpatient departments. The first physiotherapy session is arranged seven to ten days after surgery, for assessment of shoulder ROM, postoperative pain, function, arm swelling, patients' goals and assessment of confidence to carry out prescribed exercises. Participants are prescribed an individually tailored home exercise programme and provided with guidance on rehabilitation, management of postoperative complications and returning to general physical activity and/or work. The intervention targets three movement directions using a combination of active-assisted ROM, active ROM and stretches: shoulder flexion (forward), shoulder abduction (side), and abduction with external rotation (open chest). The second appointment is between four to six weeks postoperatively to review progress and prescribe shoulder strengthening exercises. The programme is progressed by increasing exercise repetitions, sets and resistance. The third appointment is recommended for between 12 to 16 weeks postoperatively, for further progression to facilitate return to work, sport and hobbies. For women with later entry on the basis of postoperative radiotherapy, these timings will be slightly delayed, but the exercise programme should commence at the earliest opportunity, thus within six weeks of surgery.

As per development work with patient representatives, and to reflect the pragmatic trial design, three additional physiotherapy consultations are available on request. The timing and delivery of additional appointments, either via telephone or face-to-face, are flexible to account for on-going treatment, physiotherapist judgement and patient preference. Ideally the intervention will be completed within the first six months following surgery, but women can contact their physiotherapist for up to 12 months after randomisation. Thus any late treatment-related shoulder problems will be dealt with by the trial physiotherapist. Number and method of physiotherapy contacts will be closely monitored during the trial.

For peer review only - http://bmjopen.bmj.com/site/about/guidelines.xhtml

Outcomes

Figure 1 and Table 3 present the study outcome measures and standardised assessment scales by assessment time point. Questionnaires are completed at baseline on recruitment, then at 6 weeks, 6 and 12 months after randomisation by post. The **primary outcome** is upper limb function at 12 months measured using the Disabilities of Arm, Shoulder and Hand (DASH) questionnaire (14). We considered other patient-reported outcome measures, including shoulder-specific scales, however selected the DASH because it captures symptoms and function of the upper limb rather than the shoulder joint *per se.* There is good evidence to suggest that women experience a variety of difficulties and restrictions after breast cancer treatment, affecting the hand, arm and shoulder. Functional impairment to the arm can affect performance of simple daily activities, including writing, opening or closing jars, lifting and/or holding shopping bags.

For peer review only

Table 3. Outcome assessment

Outcome	Domain	Scale / measure	T_0 baseline	t_1 6 weeks	t_2 6 months	t_3 12 months
Primary	Function	DASH				√
Secondary	Function	DASH subscales	√		√	√
	Acute & chronic pain	FACT-B+4; NRS DN4	√	√	√	√
	Neuropathic pain		√	√	√	√
	Complications	SSI + self-report		√	√	√
	Lymphoedema	Self-report	√	√	√	√
	Health-related QoL	SF12 / EQ-5D-5L	√		√	√
	Resource use	Self-report			√	√
	General activity & exercise	PASE items	√	√	√	√

DASH: Disabilities of Arm, Shoulder and Hand questionnaire; DN4: Doleur Neuropathique; SF12: Short Form-12; SSI: surgical site infection; EQ-5D-5L: Euroqol; t: time point; QoL: Quality of Life; PASE: Physical Activity Scale for the Elderly

For peer review only - http://bmjopen.bmj.com/site/about/guidelines.xhtml

The DASH is a 30-item patient-reported outcome measure designed to capture difficulty in performing various upper arm activities (14, 15). A single DASH score is generated, although psychometric assessment using discriminant content validation analysis has shown that the scale can be used to produce three health outcome sub-scores for impairment, activity limitation and participation restriction, as per the WHO International Classification of Functioning Disability and Health (ICF) taxonomy (16).

Secondary outcomes include health-related quality of life (EuroQol EQ-5D-5L and Short-Form-12), DASH sub-scores, and surgical adverse events including pain (acute, chronic, neuropathic pain) surgical site infection and lymphoedema. A numerical rating scale (NRS) 0-10 and Doleur Neuropathique Questionnaire (DN4) are used to collect pain intensity and pain character. The Functional Assessment of Cancer Therapy-Breast (FACT-B4) subscale captures arm tenderness, numbness, painful movement and stiffness. We added items to capture arm heaviness and swelling as self-report indicators of lymphoedema. Data on exercise/mobility are collected to allow comparisons in physical activity (selected items from the Physical Activity Scale for the Elderly (PASE)). The PASE was designed for use with older adults has been validated for use in clinical trials recruiting patients aged 55 years and older (17). Healthcare resource use is recorded for economic analyses.

For peer review only

Sample Size

The PROSPER trial aims to recruit 350 patients, allocated in a 1:1 ratio. The sample size calculation is based on a Dutch trial of thirty women with breast cancer, randomised to physiotherapy over a three month period, reporting a between group difference of 7 points on the DASH at 6 months (18). At 80% power and $p<0.05$, this yields a target of 242 participants in total. Accounting for therapist effects, an intracluster coefficient (ICC) of 0.01 (yielding a design effect of 1.05), gives a target of 256 participants. The ICC estimate is based on our previous experience of exercise interventions in a range of musculoskeletal trials. We anticipate loss to follow up of less than 10% based on our previous clinical trials

For peer review only - http://bmjopen.bmj.com/site/about/guidelines.xhtml

however, have inflated this to 25% to cover the possibility that numbers lost to follow up are greater than anticipated e.g. due to ongoing cancer treatment.

The study is powered to detect a 7 point difference on the DASH. Studies of rheumatological and orthopaedic populations have suggested that the minimally clinically important difference (MCID) for the DASH is 10, and that the between group difference for trials should be set at 10 (19). However, this fails to account for many of the eventualities that occur in pragmatic trials, notably that there is not a 'no treatment' control arm, and therefore that some of the control group may be exposed by serendipity to an intervention of similar intensity, particularly in a high risk population.

Internal pilot study

A six-month internal pilot phase was conducted at three breast cancer units (Coventry, Oxford and Wolverhampton) to evaluate processes for patient identification, eligibility and refinement of recruitment estimates. The intended sample size for the internal pilot study was 30 participants, approximately 10% of the full sample. Acceptability of the PROSPER intervention was explored through qualitative research involving audio-recorded individual interviews with seven participants. Changes were made to patient-facing materials and to exercise intervention materials. Easy to use pocket-sized laminated cards with details of inclusion/exclusion criteria and shoulder screening criteria were produced for recruitment staff. Additional telephone or face-to-face appointments were added to the exercise intervention to allow for flexibility during ongoing cancer treatment. Data from the pilot phase helped to refine recruitment and trial processes. Patients recruited to the pilot phase continue with the follow-up schedule and will be retained in the full trial analysis. The pilot study was completed as planned and the funder approved progression to full trial.

For peer review only - http://bmjopen.bmj.com/site/about/guidelines.xhtml

Data analysis

Statistical analysis will be intention-to-treat and will comply with the CONSORT guidelines. The primary outcome data will be summarised using mean, standard deviation, median and range values. The clustering effect will be assessed prior to analysis of the data. In the presence of a clustering effect, the primary outcome will be analysed using multi-level linear regression models. If there is negligible clustering effect, it will be analysed using ordinary linear regression models. In each case, the mean change from baseline (to 6 and 12 months) will be summarised for each of the treatment arms and differences between the interventions using unadjusted and adjusted (for age, type of surgery and radiotherapy) estimates. These mean changes and their 95% confidence intervals will be plotted graphically so that change can be assessed over the course of the study. Continuous secondary outcomes will be assessed in a similar way to the primary outcome. Categorical data will be analysed using random effect/ordinary logistic models, depending on the presence of a clustering effect.

A DASH score cannot be computed if there are more than three missing items. As a sensitivity analysis, the impact of missing data will be assessed using multiple imputation. The impact of non-compliance with the intervention will be examined using the complier average causal effect (CACE) analysis (20, 21). We have reviewed definitions of compliance for CACE analyses used in other therapy trials (22, 23). Complete compliance with the PROSPER intervention is defined as having three or more contacts with the PROSPER therapist; an additional analysis will be undertaken to explore partial compliance, defined as less than three sessions. Analyses and template tables will be reported in a detailed statistical analysis plan for review and approval by the DMC, prior to final statistical analysis of the data. Planned sensitivity analyses include: a) the impact of low/high recruitment centres on clustering effect; and b) assessment of differences between date of randomisation and date of surgery across groups, as surgical trials vary in relation to timing of follow-up.

For peer review only - http://bmjopen.bmj.com/site/about/guidelines.xhtml

Economic Evaluation

The primary economic evaluation will be conducted from the NHS and personal social services (PSS) perspective (24) using the intention-to-treat approach (25). Data will be collected on the health and social service resources used in the treatment of each trial participant from randomisation to 12 months post-randomisation. Primary research methods will be used to estimate the costs of delivering the physiotherapy-led exercise programme, including development and training of accredited providers, the cost of delivering the individual sessions and participant monitoring activities. Broader resource utilisation will be captured through three main sources: (i) clinical data extraction forms; (ii) patient postal questionnaires at 6 months and 12 months post-randomisation; and if feasible within the trial timeline, (iii) routine health data sources from NHS Digital. Current UK unit costs, will be applied to each resource item to estimate costs in each trial arm. Health-related quality of life will be measured at baseline and at 6 and 12 months post-randomisation using the generic EQ-5D-5L and SF-12 measures; national tariff sets will be used to generate quality-adjusted life-years (QALYs) (26-30).

An incremental cost-effectiveness analysis, expressed in terms of incremental cost per QALY gained, will be performed. Detailed methods of analysis will be pre-specified within a health economics analysis plan approved by the trial team prior to analysis to ensure appropriate methods are used. Results will be presented using incremental cost-effectiveness ratios (ICERs) and cost-effectiveness acceptability curves generated via the net-benefit framework. A series of sensitivity analyses will be undertaken to explore the implications of uncertainty on the ICERs and to consider the broader issue of the generalisability of the study results. Due to the known limitations of within-trial economic evaluations (31), a decision-analytical model may be constructed to examine the longer term costs and outcomes beyond the end of the trial. Costs and outcomes beyond the first year

For peer review only - http://bmjopen.bmj.com/site/about/guidelines.xhtml

will be discounted to present values (24) and probabilistic sensitivity analyses will be undertaken to explore the impact of uncertainty on the ICERs.

Qualitative sub-study

An embedded qualitative study will be undertaken to gain insight into the experiences of women participating in trial interventions. We will explore the acceptability of the exercise programme and compare and contrast experiences with women allocated to the control intervention.

Design of sub-study

In-depth, semi-structured interviews will be conducted and audio-recorded. Interview topic guides will be used to ensure similar areas are covered in each interview. Participants consenting to the main trial are asked to indicate willingness to take part in a future interview to explore postoperative experiences. A total of twenty interviews are planned, with ten women from each intervention arm. Purposive sampling will be used, striving for a mix of geographical location, age, employment status, socio-economic background and ethnicity.

Analysis

Interviews will be recorded, transcribed and analysed using a Framework Approach. A thematic framework will be developed using pre-determined themes plus new themes raised by participants. The framework will be applied to the interview text and coded data will be arranged on a chart according to each theme identified. Themes will be examined with a view to providing explanations of the participants' experiences and understandings.

Data security and management

Participant data is stored on a secure database in accordance with the Data Protection Act (1998). A unique trial identification number is used on all participant communication. Clinical and patient forms are being checked for completeness and congruity before data entry onto

For peer review only - http://bmjopen.bmj.com/site/about/guidelines.xhtml

the PROSPER trial database. Data will undergo additional checks to ensure consistency between data submitted and original paper forms. Trial documentation and data will be archived for at least ten years after completion of the trial in accordance with WCTU standard operating procedures.

Trial monitoring

The Trial Management Group (TMG) will oversee all aspects of design, delivery, quality assurance and data analysis. A Trial Steering Committee (TSC), with independent Chairperson, will monitor the trial at least once per year. An independent DMC will review trial progress, recruitment, protocol compliance and interim analysis of outcomes, annually or more frequently as requested. Recruitment data from the internal pilot study were reviewed by independent committees and by the funder to approve the launch of the main trial.

Adverse event management

A safety reporting protocol has been developed for related and unexpected serious adverse events (SAEs) and directly attributable adverse events (AEs). An AE is defined as any untoward medical occurrence in a subject which does not necessarily have a causal relationship with the intervention. Any adverse event that occurs whilst undertaking PROSPER exercises, either during an appointment, or whilst exercising unsupervised at home, require reporting to the trial team. The trial Chief Investigator, with input from the WCTU Quality Assurance team, determine whether AEs require reporting to the trial sponsor, DMC and Ethics Committee, in accordance with the full safety reporting protocol.

Research ethics approval

Ethical approval was granted from the NHS National Research Ethics Service (NRES) Committee West Midlands (Solihull) (15/WM/0224) on 20th July 2015. Site-specific

For peer review only - http://bmjopen.bmj.com/site/about/guidelines.xhtml

approvals have been obtained from NHS Research, Development and Innovation departments.

Dissemination policy

The study team are committed to full disclosure of the results of the trial. Findings will be reported in accordance with CONSORT guidelines (32) and we aim to publish in high impact journals. Our patient representatives will assist with dissemination of study results through INVOLVE, other cancer patient groups and organisations including www.independentcancerpatientsvoice.org.uk. The funder will take no role in the analysis or interpretation of trial results.

Discussion

The PROSPER trial will be the largest UK RCT examining the effectiveness of an early, supervised exercise and behavioural support intervention for women at risk of developing shoulder problems after breast cancer surgery. Previous trials in this field have been criticised for being of poor methodological quality and lacking in important outcome measures, such as patient-reported shoulder function and health-related quality of life. Another challenge encountered in previous clinical trials of this population is low participant recruitment, partly due to the short time frame between diagnosis and surgery but also perhaps compounded by reluctance to undertake active exercise when faced with a distressing and potentially life-threatening cancer diagnosis. PROSPER aims to recruit 350 newly diagnosed breast cancer patients to provide empirical data on whether a physiotherapy-led exercise programme is effective for reducing shoulder disability, when delivered in a pragmatic NHS clinical setting. The design and development of this complex intervention was underpinned by multiple stages of work, in line with MRC guidance on the development of complex interventions. A full description of the content of the PROSPER exercise intervention has been submitted elsewhere for publication.

For peer review only - http://bmjopen.bmj.com/site/about/guidelines.xhtml

Figure 1 Study outcome measures and assessment time points

Figure 2 Trial flow diagram

Acknowledgements

We extend very grateful thanks to all the trial participants. We are also grateful to all the physiotherapy staff, surgical oncology teams, breast cancer nurses and research departments collaborating on this study.

Collaborators

PROSPER Study Group: Chief Investigator: Professor Julie Bruce. Co-investigators (Grant holders): Professor Sarah E Lamb, Dr Esther Williamson, Dr Ranjit Lall, Professor Stavros Petrou, Mr Alastair M Thompson, Dr John Williams and Dr Catherine Harkin (deceased).

Trial Coordination/Administration: Mrs Emma J Withers, Mrs Lauren Betteley, Mr Craig Turner, Mrs Loraine Chowdhury.

Senior Project Managers: Mrs Susie Hennings, Mrs Helen Higgins.

Research Fellows/Associates: Dr Helen Richmond, Mrs Clare Lait (Physiotherapist), Dr Sophie Rees (Qualitative), Mr Pankaj Mistry (Medical Statistics), Dr Alastair Canaway (Health Economics).

Patient representatives: Dr Catherine Harkin (Deceased), Mrs Marie van Laar, Mrs Lyn Ankcorn.

Surgical Leads: Miss Abigail Tomlins, Miss Raghavan Vidya, Ms Pankaj G Roy, Miss Kat McEvoy, Miss Rachel Soulsby.

Intervention development: Mrs Clare Lait, Dr Esther Williamson, Dr Cynthia Srikesavan, Mrs Jane Moser, Dr Meredith Newman, Dr Sophie Rees, Mrs Lauren Betteley, Dr Helen Richmond, Dr Beth Fordham, Professor Sarah E Lamb and Professor Julie Bruce.

Data Programming team: Mr Ade Willis, Mr Henry Adjei.

Quality Assurance: Ms Claire Daffern.

For peer review only - http://bmjopen.bmj.com/site/about/guidelines.xhtml

Contributors: JB obtained study funding with support from SEL, EW, RL, SP and AMT. JB, SEL, EW, CL, RL, AMT, LB, SR and SP participated in the design of the study. EJW and LB coordinate study administration, acquisition of trial data and administrative support (CT/LC). PM will undertake statistical analysis, under direction of RL, senior trial statistician. AC is responsible for health economic analysis, supported by SP, senior health economist. JB and HR drafted the manuscript. All authors critically revised the manuscript for intellectual content and approved the final manuscript. This trial protocol is published on behalf of the PROSPER Study Group.

Funding

The PROSPER trial is funded by the National Institute of Health Research Technology Assessment Programme (NiHR HTA), project number 13/84/10. The views expressed in this publication are those of the authors and not necessarily those of the NIHR or Department of Health. This project benefited from facilities funded by Birmingham Science City Translational Medicine Clinical Research and Infrastructure Trials Platform, with support from Advantage West Midlands (AWM). The trial sponsor is the University of Warwick and University Hospitals Coventry and Warwickshire NHS Trust. SL and EW are supported by funding from the National Institute for Health Research (NIHR) Collaboration for Leadership in Applied Health Research and Care Oxford at Oxford Health NHS Foundation Trust, and the NIHR Biomedical Research Unit, Oxford. The views expressed are those of the author(s) and not necessarily those of the NHS, the NIHR or the Department of Health.

Trial Registration:

International Standard Randomised Controlled Trial Number: ISRCTN 35358984.

Competing interests

One author provides private physiotherapy to cancer patients (CL).

For peer review only - http://bmjopen.bmj.com/site/about/guidelines.xhtml

Data sharing statement

The trial statisticians and iDMC will have access to the dataset for the analysis of trial outcomes. The CI will have access to the data and take full responsibility for the analysis and publication of results. Once the main analyses have been undertaken, data will be available to other investigators subject to approval of data analysis plans by the steering committee and compliance with the University of Warwick Standard Operating Procedures on Data Management and Sharing. We will comply with Data Sharing Policies that may be instituted by the Funder (NIHR) during the lifetime of the project. The full PROSPER intervention manual and related materials will be available for wider access on completion of the main trial, according to funder and institutional repository requirements.

Data Monitoring Committee: Professor Malcolm Reed (Chair), Dr Rhian Gabe, Dr Matthew Maddocks.

Trial Steering Committee: Professor Steven Duffy (Chair), Dr Anna Kirby, Dr Karen Robb.

We dedicate this article to Professor Adele Frances (Deceased) who served on the PROSPER TSC from 2015-2016.

For peer review only

References

1. CRUK. *Cancer Research UK: Breast Cancer Survival Statistics* [cited 2014. Available from: http://www.cancerresearchuk.org/cancer-info/cancerstats/types/breast/survival/.

2. Mejdahl MK, Andersen KG, Gärtner R, Kroman N, Kehlet H. Persistent pain and sensory disturbances after treatment for breast cancer: six year nationwide follow-up study. BMJ. 2013;346:f1865.

3. McNeely ML, Binkley JM, Pusic AL, Campbell KL, Gabram S, Soballe PW. A prospective model of care for breast cancer rehabilitation: postoperative and postreconstructive issues. Cancer. 2012;118(8 Suppl):2226-36.

4. Lee TS, Kilbreath SL, Refshauge KM, Herbert RD, Beith JM. Prognosis of the upper limb following surgery and radiation for breast cancer. Breast Cancer Res Treat. 2008;110(1):19-37.

5. Bruce J, Thornton AJ, Powell R, Johnston M, Wells M, Heys SD, et al. Psychological, surgical, and sociodemographic predictors of pain outcomes after breast cancer surgery: a population-based cohort study. Pain. 2014;155(2):232-43.

6. Kootstra JJ, Dijkstra PU, Rietman H, de Vries J, Baas P, Geertzen JH, et al. A longitudinal study of shoulder and arm morbidity in breast cancer survivors 7 years after sentinel lymph node biopsy or axillary lymph node dissection. Breast Cancer Res Treat. 2013;139(1):125-34.

7. Meretoja TJ, Andersen KG, Bruce J, Haasio L, Sipila R, Scott NW, et al. Clinical Prediction Model and Tool for Assessing Risk of Persistent Pain After Breast Cancer Surgery. J Clin Oncol. 2017;35(15):1660-7.

8. DiSipio T, Rye S, Newman B, Hayes S. Incidence of unilateral arm lymphoedema after breast cancer: a systematic review and meta-analysis. The Lancet Oncology. 2013;14(6):500-15.

For peer review only

For peer review only - http://bmjopen.bmj.com/site/about/guidelines.xhtml

9. McNeely ML, Campbell K, Ospina M, Rowe BH, Dabbs K, Klassen TP, et al. Exercise interventions for upper-limb dysfunction due to breast cancer treatment. The Cochrane database of systematic reviews. 2010(6):CD005211.

10. Todd J, Scally A, Dodwell D, Horgan K, Topping A. A randomised controlled trial of two programmes of shoulder exercise following axillary node dissection for invasive breast cancer. Physiotherapy. 2008;94(4):265-73.

11. Chan A-W, Tetzlaff JM, Gøtzsche PC, Altman DG, Mann H, Berlin JA, et al. SPIRIT 2013 explanation and elaboration: guidance for protocols of clinical trials. Bmj. 2013;346:e7586.

12. Health Do, cartographer Toolkit for producing patient information London The Department of Health; 2003.

13. BCC. Breast Cancer Campaign [Available from: http://www.breastcancercare.org.uk/.

14. Hudak PL, Amadio PC, Bombardier C. Development of an upper extremity outcome measure: the DASH (disabilities of the arm, shoulder and hand) [corrected]. The Upper Extremity Collaborative Group (UECG). Am J Ind Med. 1996;29(6):602-8.

15. Harrington S, Michener LA, Kendig T, Miale S, George SZ. Patient-reported upper extremity outcome measures used in breast cancer survivors: a systematic review. Archives of physical medicine and rehabilitation. 2014;95(1):153-62.

16. Dixon D, Johnston M, McQueen M, Court-Brown C. The Disabilities of the Arm, Shoulder and Hand Questionnaire (DASH) can measure the impairment, activity limitations and participation restriction constructs from the International Classification of Functioning, Disability and Health (ICF). BMC musculoskeletal disorders. 2008;9:114.

17. Washburn RA, McAuley E, Katula J, Mihalko SL, Boileau RA. The physical activity scale for the elderly (PASE): evidence for validity. Journal of clinical epidemiology. 1999;52(7):643-51.

For peer review only - http://bmjopen.bmj.com/site/about/guidelines.xhtml

18. Beurskens CH, van Uden CJ, Strobbe LJ, Oostendorp RA, Wobbes T. The efficacy of physiotherapy upon shoulder function following axillary dissection in breast cancer, a randomized controlled study. BMC cancer. 2007;7:166.

19. Sorensen AA, Howard D, Tan WH, Ketchersid J, Calfee RP. Minimal clinically important differences of 3 patient-rated outcomes instruments. The Journal of hand surgery. 2013;38(4):641-9.

20. Dunn G, Maracy M, Tomenson B. Estimating treatment effects from randomized clinical trials with noncompliance and loss to follow-up: the role of instrumental variable methods. Statistical methods in medical research. 2005;14(4):369-95.

21. Perneger TV, Burnand B. A simple imputation algorithm reduced missing data in SF-12 health surveys. Journal of clinical epidemiology. 2005;58(2):142-9.

22. Knox CR, Lall R, Hansen Z, Lamb SE. Treatment compliance and effectiveness of a cognitive behavioural intervention for low back pain: a complier average causal effect approach to the BeST data set. BMC musculoskeletal disorders. 2014;15:17.

23. Williams MA, Williamson EM, Heine PJ, Nichols V, Glover MJ, Dritsaki M, et al. Strengthening And stretching for Rheumatoid Arthritis of the Hand (SARAH). A randomised controlled trial and economic evaluation. Health Technol Assess. 2015;19(19):1-222.

24. NICE. *Guide to the Methods of Technology Appraisal* 2013 [Available from: http://publications.nice.org.uk/guide-to-the-methods-of-technology-appraisal-2013-pmg9.

25. Hollis S, Campbell F. What is meant by intention to treat analysis? Survey of published randomised controlled trials. Bmj. 1999;319(7211):670-4.

26. Dolan P. Modeling valuations for EuroQol health states. Medical care. 1997;35(11):1095-108.

27. van Hout B, Janssen MF, Feng YS, Kohlmann T, Busschbach J, Golicki D, et al. Interim scoring for the EQ-5D-5L: mapping the EQ-5D-5L to EQ-5D-3L value sets. Value in health : the journal of the International Society for Pharmacoeconomics and Outcomes Research. 2012;15(5):708-15.

For peer review only

For peer review only - http://bmjopen.bmj.com/site/about/guidelines.xhtml

28. Herdman M, Gudex C, Lloyd A, Janssen M, Kind P, Parkin D, et al. Development and preliminary testing of the new five-level version of EQ-5D (EQ-5D-5L). Quality of life research : an international journal of quality of life aspects of treatment, care and rehabilitation. 2011;20(10):1727-36.

29. Brazier J, Roberts J, Deverill M. The estimation of a preference-based measure of health from the SF-36. Journal of health economics. 2002;21(2):271-92.

30. Devlin N, Shah K, Feng Y, Mulhern B, van Hout B. Valuing health-related quality of life: an EQ-5D-5L value set for England. 2016.

31. Sculpher MJ, Claxton K, Drummond M, McCabe C. Whither trial-based economic evaluation for health care decision making? Health economics. 2006;15(7):677-87.

32. Moher D, Schulz KF, Altman DG. The CONSORT statement: revised recommendations for improving the quality of reports of parallel-group randomised trials. Lancet. 2001;357(9263):1191-4.

For peer review only

For peer review only - http://bmjopen.bmj.com/site/about/guidelines.xhtml

Figure 1. Study outcome measures and assessment time points

	Study period				
	Enrolment	Allocation	Post-allocation		
**Time point**	*-t₁*	*0*	t_1 6 weeks	t_2 6 months	t_3 12 months
Enrolment:					
Eligibility screen	√				
Informed consent	√				
Randomisation	350	√			
Interventions					
Usual Care (UC)	175	All participants	←→		
UC + PROSPER intervention	175		←	—	→

Figure 1 Study outcome measures and assessment time points

87x46mm (300 x 300 DPI)

review only

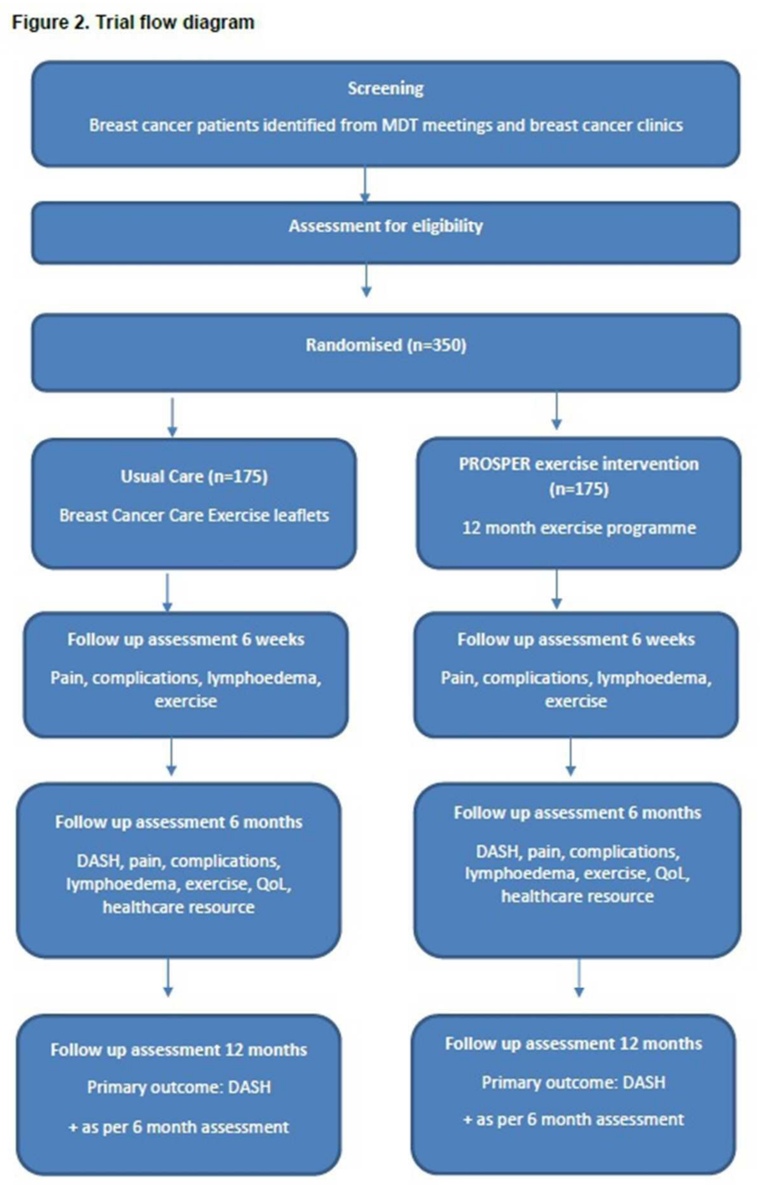

Figure 2 Trial flow diagram

50x65mm (300 x 300 DPI)

STANDARD PROTOCOL ITEMS: RECOMMENDATIONS FOR INTERVENTIONAL TRIALS

SPIRIT 2013 Checklist: Recommended items to address in a clinical trial protocol and related documents*

Section/item	Item No	Description	Addressed on page number
**Administrative information**			
Title	1	Descriptive title identifying the study design, population, interventions, and, if applicable, trial acronym	1
Trial registration	2a	Trial identifier and registry name. If not yet registered, name of intended registry	4
	2b	All items from the World Health Organization Trial Registration Data Set	Table 1
Protocol version	3	Date and version identifier	3 & 22
Funding	4	Sources and types of financial, material, and other support	25 & Table 1
Roles and responsibilities	5a	Names, affiliations, and roles of protocol contributors	24-25
	5b	Name and contact information for the trial sponsor	Table 1
	5c	Role of study sponsor and funders, if any, in study design; collection, management, analysis, and interpretation of data; writing of the report; and the decision to submit the report for publication, including whether they will have ultimate authority over any of these activities	25-6
	5d	Composition, roles, and responsibilities of the coordinating centre, steering committee, endpoint adjudication committee, data management team, and other individuals or groups overseeing the trial, if applicable (see Item 21a for data monitoring committee)	24

For peer review only - http://bmjopen.bmj.com/site/about/guidelines.xhtml

Introduction

Background and rationale	6a	Description of research question and justification for undertaking the trial, including summary of relevant studies (published and unpublished) examining benefits and harms for each intervention	5 - 7
	6b	Explanation for choice of comparators	10 – 12
Objectives	7	Specific objectives or hypotheses	8
Trial design	8	Description of trial design including type of trial (eg, parallel group, crossover, factorial, single group), allocation ratio, and framework (eg, superiority, equivalence, noninferiority, exploratory)	8 - 9

Methods: Participants, interventions, and outcomes

Study setting	9	Description of study settings (eg, community clinic, academic hospital) and list of countries where data will be collected. Reference to where list of study sites can be obtained	8
Eligibility criteria	10	Inclusion and exclusion criteria for participants. If applicable, eligibility criteria for study centres and individuals who will perform the interventions (eg, surgeons, psychotherapists)	8 – 9
Interventions	11a	Interventions for each group with sufficient detail to allow replication, including how and when they will be administered	10-12 + separate intervention papers
	11b	Criteria for discontinuing or modifying allocated interventions for a given trial participant (eg, drug dose change in response to harms, participant request, or improving/worsening disease)	22
	11c	Strategies to improve adherence to intervention protocols, and any procedures for monitoring adherence (eg, drug tablet return, laboratory tests)	12
	11d	Relevant concomitant care and interventions that are permitted or prohibited during the trial	12
Outcomes	12	Primary, secondary, and other outcomes, including the specific measurement variable (eg, systolic blood pressure), analysis metric (eg, change from baseline, final value, time to event), method of aggregation (eg, median, proportion), and time point for each outcome. Explanation of the clinical relevance of chosen efficacy and harm outcomes is strongly recommended	Table 2
Participant timeline	13	Time schedule of enrolment, interventions (including any run-ins and washouts), assessments, and visits for participants. A schematic diagram is highly recommended (see Figure)	Figure 1

For peer review only - http://bmjopen.bmj.com/site/about/guidelines.xhtml

Sample size	14	Estimated number of participants needed to achieve study objectives and how it was determined, including clinical and statistical assumptions supporting any sample size calculations	16
Recruitment	15	Strategies for achieving adequate participant enrolment to reach target sample size	9
**Methods: Assignment of interventions (for controlled trials)**			
Allocation:			
Sequence generation	16a	Method of generating the allocation sequence (eg, computer-generated random numbers), and list of any factors for stratification. To reduce predictability of a random sequence, details of any planned restriction (eg, blocking) should be provided in a separate document that is unavailable to those who enrol participants or assign interventions	9
Allocation concealment mechanism	16b	Mechanism of implementing the allocation sequence (eg, central telephone; sequentially numbered, opaque, sealed envelopes), describing any steps to conceal the sequence until interventions are assigned	9
Implementation	16c	Who will generate the allocation sequence, who will enrol participants, and who will assign participants to interventions	10
Blinding (masking)	17a	Who will be blinded after assignment to interventions (eg, trial participants, care providers, outcome assessors, data analysts), and how	10
	17b	If blinded, circumstances under which unblinding is permissible, and procedure for revealing a participant's allocated intervention during the trial	10
**Methods: Data collection, management, and analysis**			
Data collection methods	18a	Plans for assessment and collection of outcome, baseline, and other trial data, including any related processes to promote data quality (eg, duplicate measurements, training of assessors) and a description of study instruments (eg, questionnaires, laboratory tests) along with their reliability and validity, if known. Reference to where data collection forms can be found, if not in the protocol	11 & Table 2
	18b	Plans to promote participant retention and complete follow-up, including list of any outcome data to be collected for participants who discontinue or deviate from intervention protocols	11 & 14

For peer review only - http://bmjopen.bmj.com/site/about/guidelines.xhtml

Data management	19	Plans for data entry, coding, security, and storage, including any related processes to promote data quality (eg, double data entry; range checks for data values). Reference to where details of data management procedures can be found, if not in the protocol	20
Statistical methods	20a	Statistical methods for analysing primary and secondary outcomes. Reference to where other details of the statistical analysis plan can be found, if not in the protocol	17-20
	20b	Methods for any additional analyses (eg, subgroup and adjusted analyses)	17-20
	20c	Definition of analysis population relating to protocol non-adherence (eg, as randomised analysis), and any statistical methods to handle missing data (eg, multiple imputation)	18-20
**Methods: Monitoring**			
Data monitoring	21a	Composition of data monitoring committee (DMC); summary of its role and reporting structure; statement of whether it is independent from the sponsor and competing interests; and reference to where further details about its charter can be found, if not in the protocol. Alternatively, an explanation of why a DMC is not needed	22
	21b	Description of any interim analyses and stopping guidelines, including who will have access to these interim results and make the final decision to terminate the trial	20
Harms	22	Plans for collecting, assessing, reporting, and managing solicited and spontaneously reported adverse events and other unintended effects of trial interventions or trial conduct	22
Auditing	23	Frequency and procedures for auditing trial conduct, if any, and whether the process will be independent from investigators and the sponsor	12 & described in intervention papers
**Ethics and dissemination**			
Research ethics approval	24	Plans for seeking research ethics committee/institutional review board (REC/IRB) approval	22
Protocol amendments	25	Plans for communicating important protocol modifications (eg, changes to eligibility criteria, outcomes, analyses) to relevant parties (eg, investigators, REC/IRBs, trial participants, trial registries, journals, regulators)	4

For peer review only - http://bmjopen.bmj.com/site/about/guidelines.xhtml

Consent or assent	26a	Who will obtain informed consent or assent from potential trial participants or authorised surrogates, and how (see Item 32)	9
	26b	Additional consent provisions for collection and use of participant data and biological specimens in ancillary studies, if applicable	9 & 21
Confidentiality	27	How personal information about potential and enrolled participants will be collected, shared, and maintained in order to protect confidentiality before, during, and after the trial	9
Declaration of interests	28	Financial and other competing interests for principal investigators for the overall trial and each study site	24
Access to data	29	Statement of who will have access to the final trial dataset, and disclosure of contractual agreements that limit such access for investigators	26
Ancillary and post-trial care	30	Provisions, if any, for ancillary and post-trial care, and for compensation to those who suffer harm from trial participation	12
Dissemination policy	31a	Plans for investigators and sponsor to communicate trial results to participants, healthcare professionals, the public, and other relevant groups (eg, via publication, reporting in results databases, or other data sharing arrangements), including any publication restrictions	3-4 & 20
	31b	Authorship eligibility guidelines and any intended use of professional writers	3
	31c	Plans, if any, for granting public access to the full protocol, participant-level dataset, and statistical code	24
**Appendices**			
Informed consent materials	32	Model consent form and other related documentation given to participants and authorised surrogates	Can be provided as Appendix if requested
Biological specimens	33	Plans for collection, laboratory evaluation, and storage of biological specimens for genetic or molecular analysis in the current trial and for future use in ancillary studies, if applicable	Not applicable

*It is strongly recommended that this checklist be read in conjunction with the SPIRIT 2013 Explanation & Elaboration for important clarification on the items. Amendments to the protocol should be tracked and dated. The SPIRIT checklist is copyrighted by the SPIRIT Group under the Creative Commons "Attribution-NonCommercial-NoDerivs 3.0 Unported" license.

For peer review only - http://bmjopen.bmj.com/site/about/guidelines.xhtml
